# Quantitative genetics of the use of conspecific and heterospecific social cues for breeding site choice

**DOI:** 10.1111/evo.14071

**Published:** 2020-08-13

**Authors:** Jere Tolvanen, Sami M. Kivelä, Blandine Doligez, Jennifer Morinay, Lars Gustafsson, Piter Bijma, Veli‐Matti Pakanen, Jukka T. Forsman

**Affiliations:** ^1^ Department of Ecology and Genetics University of Oulu Oulu 90014 Finland; ^2^ Department of Zoology, Institute of Ecology and Earth Sciences University of Tartu Tartu 51014 Estonia; ^3^ Current Address: Department of Ecology and Genetics University of Oulu Oulu 90014 Finland; ^4^ Laboratoire de Biométrie et Biologie Evolutive CNRS UMR 5558 Université de Lyon ‐ Université Claude Bernard Lyon 1 Villeurbanne 69622 France; ^5^ Department of Ecology and Genetics/Animal Ecology Uppsala University Uppsala SE‐75236 Sweden; ^6^ Animal Breeding and Genomics Wageningen University Wageningen 6700AH The Netherlands; ^7^ Department of Biological and Environmental Sciences University of Gothenburg Gothenburg SE‐40530 Sweden; ^8^ Current Address: Department of Biological and Environmental Sciences University of Gothenburg Gothenburg SE‐40530 Sweden; ^9^ Current Address: Natural Resources Institute Finland University of Oulu Oulu 90014 Finland

**Keywords:** Evolutionary potential, heritability, quantitative genetic mixed “animal” models, repeatability, social environment, social information

## Abstract

Social information use for decision‐making is common and affects ecological and evolutionary processes, including social aggregation, species coexistence, and cultural evolution. Despite increasing ecological knowledge on social information use, very little is known about its genetic basis and therefore its evolutionary potential. Genetic variation in a trait affecting an individual's social and nonsocial environment may have important implications for population dynamics, interspecific interactions, and, for expression of other, environmentally plastic traits. We estimated repeatability, additive genetic variance, and heritability of the use of conspecific and heterospecific social cues (abundance and breeding success) for breeding site choice in a population of wild collared flycatchers *Ficedula albicollis*. Repeatability was found for two social cues: previous year conspecific breeding success and previous year heterospecific abundance. Yet, additive genetic variances for these two social cues, and thus heritabilities, were low. This suggests that most of the phenotypic variation in the use of social cues and resulting conspecific and heterospecific social environment experienced by individuals in this population stems from phenotypic plasticity. Given the important role of social information use on ecological and evolutionary processes, more studies on genetic versus environmental determinism of social information use are needed.

Animals face multiple fitness‐related decisions that they must make adaptively to maximize fitness. To reduce uncertainty in decision‐making, animals need information about environmental quality (Dall et al. 2005; Schmidt et al. 2010). Such information can be obtained by observing the location, behavior, and success of other individuals, including heterospecifics (social information; Danchin et al. 2004; Seppänen et al. 2007; Schmidt et al. 2010). Cueing on others may provide high‐quality information on the environment more quickly and with more limited costs than collecting information by personally interacting with the environment (personal information; Giraldeau et al. 2002; Goodale et al. 2010). Consequently, the use of social information for decision‐making has been observed in a wide variety of contexts including foraging (Galef and Giraldeau 2001), breeding (Szymkowiak 2013), predator avoidance (Griffin 2004), and mate choice (Galef and White 2000), in diverse taxa such as birds (Slagsvold and Wiebe 2011), fish (Laland et al. 2011), and insects (Chittka and Leadbeater 2005). The use of conspecific and heterospecific social information may have major ecological and evolutionary implications for individuals, populations, and communities (Danchin et al. 2004; Seppänen et al. 2007; Schmidt et al. 2010; Gil et al. 2018). For example, by enabling individuals to assess their environment more quickly and/or at larger spatial scales compared to personal information, the use of social information may enhance individuals’ ability to respond to environmental changes, such as climate change (Ponchon et al. 2015; Keith and Bull 2017; but see Parejo 2016). The use of social information may also result in cultural evolution that may in turn affect genetic evolution and even speciation (Danchin et al. 2004; Heyer et al. 2005; Laland et al. 2010; Verzijden et al. 2012; Aplin et al. 2015).

Cueing on others often results in attraction to or avoidance of other individuals, thus the use of social information affects the social environment (i.e., type and dynamics of social interactions) that an individual experiences. For example, the use of the presence and/or success of breeding conspecifics as social cues leads individuals to be attracted to areas of higher conspecific breeding density and success (e.g., Deutsch and Nefdt 1992; Doligez et al. 2002; Sergio and Penteriani 2005; Nocera et al. 2006; Cote and Clobert 2007; Boulinier et al. 2008), where competition is thereby increased (Doligez et al. 2003). Migratory birds also use the information acquired by resident birds during the nonbreeding season by breeding preferentially in their vicinity (Mönkkönen et al. 1990; Forsman et al. 2002; Parejo et al. 2005; Sebastián‐Gonzáles et al. 2010; Kivelä et al. 2014). Thereby, the use of social cues for breeding site choice not only determines the available resources and threats for the breeding adults and their offspring but also shapes their social environment during the breeding season, which can affect the expression of many other traits both in adults and offspring, with potentially long‐lasting effects on future life and individual fitness (Pärt 2001; Sergio et al. 2009).

Despite the major ecological and evolutionary implications of the use of social information, very little is known about the genetic basis of this behavior. Consequently, its evolutionary potential remains poorly understood. Genetic variation in a trait that affects an individual's social and nonsocial environment (i.e., a niche‐constructive trait; Saltz and Nuzhdin 2014) may generate genotype‐environment correlations, that is, different genotypes experience different (social and nonsocial) environments (Saltz 2011). Thus, genetic variation in a niche‐constructive trait may also result in spatially variable selection regimes within a population that may contribute to the maintenance of genetic variation both in the niche‐constructive and other traits (Saltz and Nuzhdin 2014). Furthermore, by generating variation in environmental conditions among individuals, genetic variation in niche‐constructive traits may also have important implications for the expression of other, environment‐dependent traits (Saltz and Nuzhdin 2014; Saltz 2017). For example, in *Drosophila melanogaster*, genetic variation in group size preference led to variation in social interactions among individuals that in turn influenced the development of another social behavior, namely, aggressiveness toward conspecifics (Saltz 2017).

Previous work with laboratory populations suggests that genetic variation contributes to among‐individual variation in the use of conspecific social information and choice of social environment in *Drosophila* fruit flies (Saltz 2011; Foucaud et al. 2013; Philippe et al. 2016; Geiger and Saltz 2020). The choice of conspecific social environment (colony size) was also found to be heritable in the colonial cliff swallow (*Petrochelidon pyrrhonota*) (Brown and Brown 2000). Whether these results can be generalized to other social cues and/or organisms in the wild, however, remains uncertain. Previous studies on other social behaviors have reported genetic variation in some traits (e.g., cooperative breeding: Charmantier et al. 2007; received aggression and affiliative behavior: Lea et al. 2010; affiliative behavior and initiated aggression: Brent et al. 2013; social dominance, aggression: Weiss and Foerster 2013), but not in others (initiated aggression and affiliative behavior: Lea et al. 2010; received aggression: Brent et al. 2013), sometimes within the same populations. Furthermore, for a given social behavior, genetic variation may also vary considerably across species (e.g., aggressiveness: Lea et al. 2010; Brent et al. 2013; Weiss and Foerster 2013).

The social environment experienced by an individual includes not only conspecifics but also heterospecifics, for example, through competition for shared resources, but also as valuable information sources (e.g., Seppänen et al. 2007; Goodale et al. 2010; Farine et al. 2015). Genetic variation in the use of heterospecific social cues or the choice of heterospecific social environment may have similar ecological and evolutionary implications as conspecific social cues or environment (see Saltz 2011; Saltz and Nuzhdin 2014). Yet, genetic variation in heterospecific social environment choice can also have specific consequences as it may create varying neighborhood structures within populations with certain individuals associating more often with heterospecifics than others. Such variation in interspecific interactions may have important effects on population dynamics and information transmission within and between species (Seppänen et al. 2007; Goodale et al. 2010; Gil et al. 2018). For example, higher abundance of heterospecifics in the neighborhood relative to conspecifics may result in individuals preferring to use heterospecific compared to conspecific information (Jaakkonen et al. 2015; Firth et al. 2016). Therefore, it would be particularly important to assess the genetic variation in traits directly affecting interspecific interactions, such as the choice of heterospecific social environment.

Here, we investigated the quantitative genetics of the use of social information for breeding site choice. More specifically, we aimed to estimate additive genetic variance and heritability in the use of conspecific and heterospecific social cues (abundance and breeding success) for breeding site choice. Our model species was a migratory passerine, the collared flycatcher (*Ficedula albicollis*), which uses the presence and success of both conspecifics and ecologically close heterospecific competitors (e.g.; great tits *Parus major*) as social information sources for making breeding decisions at different temporal and spatial scales (Doligez et al. 1999, 2002; Seppänen and Forsman 2007; Kivelä et al. 2014; Jaakkonen et al. 2015; Morinay et al. 2020a,b). We measured the use of social cues as the difference between the abundance (or success) of conspecifics (or heterospecifics) in the neighborhood of the site chosen by a breeding pair and the average abundance (or success) of conspecifics (or heterospecifics) in the neighborhood of all available breeding sites. These metrics of social information use describe attraction to or avoidance of sites with high/low local con‐ or heterospecific abundance or success within forest patches (small scale breeding site choice). By combining these metrics of social information use with long‐term pedigree information of the studied population, we estimated the additive genetic variance and heritability in the use of these social cues.

Partitioning phenotypic variance in a trait between additive genetic and other between‐individual variance components is relevant only if the trait is repeatable, that is, shows some degree of consistent differences between individuals. Although between‐individual variance and repeatability of a trait do not directly inform about the level of additive genetic variance and heritability, they may indicate the upper limits of additive genetic variance and heritability of the trait (Nagakawa and Schielzeth 2010, but see Dohm 2002). Therefore, we first estimated individual repeatability in the use of each social cue for breeding site choice, and then partitioned between‐individual variation into additive genetic, dominance genetic, and other between‐individual variation only for those cues that showed repeatability.

Breeding site choice constitutes a phenotype jointly expressed by both the female and the male in a pair. This complicates the estimation of trait heritability, because the phenotype of a breeding pair is influenced by the additive genetic values of both breeding mates, or conversely the effect of an individual's additive genetic value not only affects its own phenotype but also affects the phenotype of its breeding mate. Thus, the level of additive genetic variation in a population may be greatly underestimated if only one sex is considered (Moore et al. 1997; Wolf et al. 1998; Wolf 2003; Bijma et al. 2007a; Bijma 2011). Furthermore, the presence of cross‐sex additive genetic covariance may enhance or impede the response to selection compared to that expected due to additive contributions of female and male additive genetic variances only (the exact effect depending on the sign and magnitude of the covariance and sex‐specific selection regimes; Chippindale et al. 2001; Foerster et al. 2007; Brommer and Rattiste 2008; Poissant et al. 2010). Therefore, we modeled breeding site choice at the breeding pair level, but estimated the between‐individual variance and individual repeatability (repeatability models) and additive genetic variance and heritability (genetic models) for each sex separately. In the genetic models, we also estimated the cross‐sex additive genetic covariance.

## Material and Methods

### DATA COLLECTION

The study area was located in the southern part of the island of Gotland, Sweden (57.00°–57.09° N, 18.29°–18.35° E) and consisted of 15 discrete deciduous forest patches interspersed with agricultural areas and/or roads. In each forest patch, nest boxes have been provided in excess since 1980 (on average about one third of the nest boxes remain unoccupied each year [Morinay et al. 2018]; for further information about the study area and patch‐specific descriptions, see Supporting Information, Section A). The collared flycatcher population breeding in nest boxes has been intensively monitored each year by visiting nest boxes at least every four days during the breeding season to record occupancy, nest building stage, timing of egg laying, clutch size, hatching date, brood size, and fledging success. All nestlings in boxes have been ringed and breeding adults have been captured (females during incubation, males during nestling rearing), ringed (or identified if already ringed), and aged as yearlings or older based on plumage characteristics (Svensson 1992). Extensive ringing of successfully breeding individuals coupled with relatively high return rates to the study area (on average, 11% in juveniles and 39% in adults; Kruuk et al. 2001) has enabled the construction of a social pedigree. Approximately 15% of nestlings originate from extra‐pair copulations in this population (Sheldon and Ellegren 1999), but such level of paternity misassignment in the social pedigree is unlikely to result in a strong bias in heritability estimates (Charmantier and Réale 2005; Firth et al. 2015).

Male collared flycatchers arrive at the breeding grounds first, a few days before females, and defend and compete over breeding sites against other males (Pärt and Qvarnström 1997). Females then choose their breeding sites from the sample of sites defended by the males. The female choice may thus be based on both the qualities of the male and environmental conditions (Qvarnström et al. 2000; Robinson et al. 2012). Breeding in the exact same nest box between years is rare, but males return to the same forest patch more often than females (Pärt and Gustafsson 1989). Nevertheless, breeding dispersal between patches is frequent in both females and males (Doligez et al. 1999)

Since 2004, breeding great tits have been monitored intensively in the same area. Great tits share main resource needs (breeding sites and food), and therefore compete with collared flycatchers (Gustafsson 1987; Forsman et al. 2008); they thus are also important social information sources for flycatchers (e.g., Seppänen and Forsman 2007; Forsman et al. 2008; Kivelä et al. 2014; Jaakkonen et al. 2015; Morinay et al. 2020a,b). Our phenotypic data on the use of social cues for breeding site choice were collected in the 15 forest patches during 2005–2010. The collared flycatcher pedigree was based on the whole flycatcher study period (1980–2010).

### DATA PREPARATION

#### Response variables

Our goal was to estimate additive genetic variance and heritability of the use of current and past (previous year) abundance and breeding success of conspecifics and heterospecifics (great tits) by collared flycatcher for breeding site (nest box) choice. The use of these social cues was assessed previously in the same population (Kivelä et al. 2014) and we derived here the response variables describing breeding site choice using the same methodology. We retained the five social cues that Kivelä et al. (2014) found to be important for nest site selection in this population, and accordingly derived five response variables, each describing the use of one of the five social cues for breeding site choice: (a) the relative abundance of conspecifics around the chosen breeding site in the current year, (b) the relative abundance of conspecifics in the previous year, (c) the relative breeding success (number of fledglings) of conspecifics in the previous year, (d) the relative abundance of great tits in the current year, and (e) the relative abundance of great tits in the previous year. Each of these response variables was calculated as the difference between the number or success of neighbors (in the current or previous year) within a specified spatial range around the nest box chosen by the focal flycatcher pair (see below) and the average number or success of neighbors (in the current or previous year) over all available (i.e., empty, including the chosen one) nest boxes in the forest patch on the day of breeding site choice (i.e., the expected number or success of neighbors for a random breeding site choice; see details in Supporting Information, Section B). The social cues were therefore included in the response variables. To account for potential intrinsic preferences of collared flycatchers for certain breeding sites (small scale habitat), the expected value of the neighborhood under random breeding site choice was calculated as a weighted average, with each nest box being weighted by its probability of occupancy by collared flycatchers during the period 1990–2000 (see Supporting Information, Section B). Positive values of the response variables indicate attraction to breeding sites surrounded by higher‐than‐average conspecific or heterospecific abundance or success, during either the current or the previous year. Conversely, negative values of the response variables indicate avoidance of such sites, and zero values indicate random breeding site choice relative to the social cue considered. Although conspecific and heterospecific abundance and success also likely reflect actual habitat quality (e.g., food abundance) and the response variables may therefore partly reflect flycatchers’ response to actual habitat quality, these and other social cues have been experimentally shown to be important for flycatcher breeding site choice in this population (Doligez et al. 2002; Seppänen and Forsman 2007; Forsman et al. 2008; Jaakkonen et al. 2015; Morinay et al. 2020a,b). The response variables here should therefore also describe the use of social cues for breeding site choice.

Kivelä et al. (2014) varied the spatial scale (i.e., neighborhood area) over which the value of each social cue was calculated, ranging from the immediate neighborhood of the nest box up to the whole breeding patch, and examined the spatial scale at which each of the cues was most likely to be used. Here, we used the most relevant spatial scale identified by Kivelä et al. (2014) for each social cue to calculate our response variables (see Table 1). Our dataset here was an extended version of the dataset used by Kivelä et al. (2014) (10 forest patches in 2005–2010). We therefore reanalyzed the use of these social cues for flycatcher breeding site choice at the spatial scales identified to be most relevant by Kivelä et al. (2014) over the extended dataset; these analyses confirmed previous results, that is, collared flycatchers used the social cues considered at the spatial scales previously reported for breeding site choice over the extended dataset (Fig. S2 in Supporting Information, Section C). The spatial scale over which the value of the social cue was calculated was defined by the parameter *α* (in meters) that was used for calculating the weights assigned to the social cues (abundance or breeding success of conspecific or heterospecific neighbors) for each nest box *j* depending on its distance to the focal nest box *i*. Only the nest boxes *j* that were within the same forest patch as the focal nest box *i* were considered, thus restricting the calculation of social cues to intra‐patch level. The weights were calculated as $e^{-(d_{ij}/\alpha )}$, where *d_ij_* is the distance between nest boxes *i* and *j* in meters (*j =* 1, …, *n_k_*; *n_k_* being the number of nest boxes in forest patch *k*; see Supporting Information for details). These weights thus exponentially decrease with increasing distance between the nest boxes, the rate of decrease being negatively correlated with the value of *α*. Hence, larger values of *α* mean that nest boxes over a larger neighborhood get a nonnegligible weight in the calculation of the social cue, or, in other words, that the social environment considered extends farther away from the focal nest box. A large value of *α* corresponds to a neighborhood including all or most nest boxes in the forest patch, and the maximum investigated value of *α*, 83 m, indeed leads to nonnegligible weight for nest boxes up to 248 m from the focal nest box. Conversely, with a small value of *α*, the neighborhood becomes essentially restricted to the few closest nest boxes (within about 20–30 m of the focal nest box), and the minimum investigated value of *α*, 1 m, includes only the focal nest box. To facilitate comparisons of the variance component estimates across social cues and to maintain consistency with the earlier phenotypic analysis (Kivelä et al. 2014), we normalized each response variable separately by dividing it by the square root of its mean square:

(1) $$y_{i}=\frac{Y_{i}}{\sqrt{\frac{\sum_{i=1}^{n}Y_{i}^{2}}{n}}},$$

where *Y_i_* is the original response variable and *i* = 1, …, *n*, *n* referring to the number of observations for the response variable.

**Table 1:** List of the response variables (social cues considered), sample sizes *n* (number of breeding pairs for which the response variable could be calculated), and the spatial scales of the neighborhood (*α* value and the respective maximum distance [radius] in meters where the weight of a nest box is >0.05; see text for details) used for each social cue

		Spatial scale of the neighborhood	
Social cue	*n*	*α*	Radius
(a) Conspecific abundance in the current year	1432	7	20
(b) Conspecific abundance in the previous year	1430	1	2
(c) Conspecific success in the previous year	1395	83	248
(d) Great tit abundance in the current year	1430	11	32
(e) Great tit abundance in the previous year	1446	75	224

#### Fixed effects

Individual's age (yearling vs. older) and, for older adults, breeding dispersal status (i.e., whether the individual bred in the same forest patch as in the previous year or not) may affect access to information sources and thus the possibilities of using social information (e.g., Doligez et al. 2002; Kivelä et al. 2014; Morinay et al. 2018, 2020b). Yearlings have decreased access to social cues in the previous breeding season, because they can collect information only after they have fledged (i.e., late in the season), and philopatric individuals may have better access to social cues in the previous year compared to immigrants. Timing of breeding also influences the availability and thus the potential use of social cues for breeding site choice (e.g., Seppänen and Forsman 2007; Kivelä et al. 2014; Jaakkonen et al. 2015). For example, the earlier settling breeders have less (if any) conspecifics breeding in the area and thus have less possibilities to use conspecific abundance as a social cue for breeding site choice compared to later breeders. To accurately analyze the use of social cues, we controlled for the variation in accessibility to different cues by including in the analyses the age and dispersal status of the breeding birds and the estimated date of initiation of nest building (i.e., the date of breeding site choice) as fixed effects. Following Kivelä et al. (2014), individual's age and dispersal status were combined into a single three‐level “status” variable: (1) yearlings, (2) older philopatric individuals (i.e., individuals that bred in the same patch as in the previous year), and (3) older immigrant individuals (i.e., older individuals that did not breed in the current patch in the previous year). To accurately account for the variation in access to social cues and also to account for potential interactions in breeding site choice between the female and the male of a pair, we also fitted all two‐ and three‐way interactions of female and male “status” and choice date variables. Our definition of the total phenotypic variance (*V*_P_, see below) excluded the variation explained by the fixed effects, thus our estimates of repeatability and heritability are conditional on the fixed effects. However, in addition to affecting the *access* to social cues, age, dispersal status, and timing of breeding may also influence the *use* of the cues. For example, yearlings lack breeding experience and philopatric individuals benefit from familiarity with their forest patch that may influence their propensity to use social cues, and earlier settling breeders may use already available heterospecific social cues more often than later settling breeders that also have conspecific cues available (Jaakkonen et al. 2015). This variation should preferably be included in the *V*_P_, but as we could not define the proportion of fixed effect variation due to variation in the *use* of cues versus in the *access* to cues, our definition of *V*_P_ excludes both. We, however, also report results based on models including only the intercept as a fixed effect in Supporting Information (Section G). We note that the results were very similar irrespective of the fixed effects structure, thus the above issue in defining the *V*_P_ unlikely affects the accuracy of the repeatability and heritability estimates. The analyses were restricted to the pairs for which the “status” variable could be defined for both female and male. In addition, some response variables had missing values, resulting in varying sample sizes (Table 1).

#### Pedigree

The full collared flycatcher pedigree was pruned to keep all individuals that had a phenotypic value for at least one response variable and their ancestors. This pruned pedigree contained 3557 individuals of which 2019 were phenotyped. Out of these phenotyped individuals, for 685 individuals both parents were known, for 61 only the mother was known, for nine only the father was known, and for 1264 both parents were unknown. These 1264 individuals were either parents of other phenotyped individuals, thus providing information for estimating the additive genetic and dominance genetic parameters, or individuals whose relatedness to other individuals was not known. In the latter case, although they did not provide information for the estimation of the additive genetic and dominance genetic parameters, they are included in the estimation of the other model components. Mean female‐male relatedness across breeding pairs (2*k*_mean_; see below for calculation) was 0.00086. Further details on the pedigree are given in the Supporting Information, Section D.

### STATISTICAL ANALYSES

#### Repeatability analyses

We estimated individual repeatability of the use of social cues using univariate linear mixed models. Phenotypic variation within each response variable was partitioned into the following components:

(2) $$y=X\beta +Z_{1}PI_{♀}+Z_{2}PI_{♂}+Z_{3}\mathrm{Patch}+Z_{4}\mathrm{Box}+e,$$

where **y** is a vector of the observed values of the response variable, and **X** and **Z** *_i_* (*i* = 1, …, 4) are design matrices relating individual observations to fixed and random effects, respectively. **β** is a vector of fixed effects, and **PI_♀_**, **PI_♂_**, **Patch**, **Box**, and **e** are vectors of random permanent individual (female and male), breeding patch, nest box effects, and residuals, respectively. The random effects and residuals were assumed to follow normal distributions with zero mean and variances *V*_PI♀_, *V*_PI♂_, *V*_PATCH_, *V*_BOX_, and *V*_R_, respectively. Less than 2% of adult collared flycatchers in our population mate with the same partner over years (Morinay et al. 2018; see also Pärt and Gustafsson 1989) making it possible to estimate permanent individual effects for females and males separately. We did not fit a population‐level **PI_♀_** – **PI_♂_** covariance, because it is technically difficult (requires specifying an “association” matrix of all individuals that is proportional to the covariance between individuals in all permanent individual effects, many of which may not be known). Thus, we assume the covariance to be zero, but also note that, given the overall low *V*_PI♀_ and *V*_PI♂_ estimates (see Results), the covariances are also likely to be low and ignoring them should not affect the conclusions of this study. The random effects **Patch** and **Box** were included to account for potential consistent spatial variation between forest patches and nest boxes, respectively. They were fitted as random effects instead of fixed effects due to the high number of levels in both variables (15 forest patches and 846 nest boxes). Fixed effects **β** included the female and male “status” variables, date of initiation of nest building, and all their two‐ and three‐way interactions.

The total phenotypic variance for the use of social cues for breeding site choice, conditioned on the fixed effects, was estimated as

(3) $$V_{P}=V_{PI♀}+V_{PI♂}+V_{PATCH}+V_{BOX}+V_{R}.$$

Female and male repeatabilities were estimated as

(4a) $$R_{♀\;}=V_{PI♀}/V_{P},$$

(4b) $$R_{♂\;}=V_{PI♂}/V_{P}.$$

The total permanent individual variance in the population was estimated as the sum of female and male permanent individual variances:

(5) $$V_{PI\;total}=V_{PI♀}+V_{PI♂}$$

and the corresponding total repeatability in the population

(6) $$R_{\mathrm{total}\;}=V_{PI\;total}/V_{P}.$$

As repeatability estimates and their 95% confidence intervals are always positive, we cannot, for example, use the criterion “95% CI excludes zero” to define which response variables are repeatable. We instead include in the following analyses those response variables for which the posterior estimates of total repeatability *R*_total_ were distributed away from zero.

#### Quantitative genetic analyses

For repeatable response variables (see Results), we estimated additive genetic variances (and heritabilities) using univariate “animal models” (quantitative genetic mixed models; Lynch and Walsh 1998; Kruuk 2004; Wilson et al. 2010).

Phenotypic variation for each response variable was partitioned into the following components:

(7) $$\begin{array}{ccc} y & = & X\beta +Z_{5}a_{♀}+Z_{6}a_{♂}+Z_{7}\mathrm{Do}m_{♀}+Z_{8}\mathrm{Do}m_{♂}+Z_{9}PI_{♀}\\ & & +\;Z_{10}PI_{♂}+Z_{11}\mathrm{Patch}+Z_{12}\mathrm{Box}+e, \end{array}$$

where, in addition to the same parameters as in the repeatability analysis (see above), **Z** *_i_* (*i* = 5, …, 8) are design matrices relating individual observations to female and male additive genetic (**a_♀_** and **a**_♂_) and dominance genetic (**Dom**_♀_ and **Dom**_♂_) random effects. The female and male additive genetic random effects were assumed to be jointly distributed following a multivariate normal distribution, MVN(**0**, **C**_A_ ⊗ **A**), where

$$C_{A}=\left[\begin{array}{cc} V_{A♀} & \mathrm{Co}v_{A♀♂}\\ \mathrm{Co}v_{A♀♂} & V_{A♂} \end{array}\right],$$

**A** is the additive genetic relationship matrix between all individuals calculated from the pruned pedigree, and ⊗ denotes the Kronecker product of the two matrices. *V*_A♀_ and *V*_A♂_ are the female and male additive genetic variances and Cov_A♀♂_ is the cross‐sex additive genetic covariance. Information for estimating female additive genetic variance comes from the covariance between *y*‐values and relatedness of females, information for estimating male additive genetic variance comes from the covariance between *y*‐values and relatedness of males, and information for female‐male additive genetic covariance comes from the covariance between *y*‐values and relatedness between females and males. The female‐male additive genetic covariance thus is related to covariance between related females and males at the population level, not necessarily between a female and a male in a breeding pair (that are typically unrelated in this population, 2*k*_mean_ = 0.00086, see above). **Dom_♀_** and **Dom_♂_** were assumed to follow normal distributions N(**0**, **D** *V*_DOM♀_) and N(**0**, **D** *V*_DOM♂_), where **D** is the dominance genetic relationship matrix between individuals, calculated from the pruned pedigree. We explicitly accounted for the dominance genetic variance because it may have a relatively important effect in natural populations (Wolak and Keller 2014). If ignored, the dominance genetic variance may not completely be subsumed to permanent individual variances **PI_•_**, but may partially confound with additive genetic variance, thus inflating the additive genetic variance estimates (Wilson et al. 2010; but see also Class and Brommer 2020 for an example of negligible effects of ignoring dominance genetic variance). Here, we tested this by fitting models excluding the dominance genetic effects and observed small increases in additive genetic variances compared to models including the dominance genetic effects (see the comparison between Tables S7 and S8 in Supporting Information, Section G). We also fitted models including the **Dom_♀_** – **Dom_♂_** covariance, but the covariance was estimated to be zero for both response variables (results not detailed). Inclusion of **PI_•_** is imperative in “animal models” if the dataset includes repeated measures of individuals (Wilson et al. 2010). As we did not find clear differences in additive genetic variance between females and males (see Results), we also fitted models constraining female and male additive genetic variances to be equal (using the linking function *mm* instead of *str* for the *MCMCglmm* call in R package MCMCglmm; Hadfield 2010). The overall result regarding the population level additive genetic variance and heritability was qualitatively identical (results not detailed) to the more detailed sex‐specific models, and thus we report the results based on the sex‐specific models.

The total phenotypic variance, conditioned on the fixed effects, was estimated as

(8) $$\begin{array}{ccc} V_{P} & = & V_{A♀}+V_{A♂}+2k_{mean}\left(2Cov_{A♀♂}\right)+V_{DOM♀}+V_{DOM♂}\\ & & +\;V_{PI♀}+V_{PI♂}+V_{PATCH}+V_{BOX}+V_{R}, \end{array}$$

where 2*k*_mean_ is the mean female‐male relatedness across breeding pairs, calculated as twice the mean pairwise kinship coefficient (*k*_mean_, derived from the pedigree) between pair members (Bijma et al. 2007a,b; Bergsma et al. 2008). Female and male narrow‐sense heritabilities were estimated as

(9a) $$h_{♀\;}^{2}=V_{A♀}/V_{P},$$

(9b) $$h_{♂\;}^{2}=V_{A♂}/V_{P}.$$

When the phenotypic value of an individual is influenced by the additive genetic effects of multiple individuals (e.g., both the female and the male pair members for breeding site choice), the population‐level estimate of additive genetic variance, which determines the potential of the trait to respond to selection, needs to include the genetic effects of both individuals (Bijma 2011). This is similar to the situation with direct and indirect genetic effects (Bijma et al. 2007a,b; Bergsma et al. 2008), but in the present case there is no distinction between focal individual and social partner, because breeding site choice is a single joint phenotype of both partners. Because response to selection depends on the change in the mean of *A*_♀_ + *A*_♂_, the total additive genetic variance equals the sum of the variance of the female trait, the male trait, and twice their covariance (Bijma 2011). Hence, we estimated the total additive genetic variance as

(10) $$V_{A\;total}=V_{A♀}+V_{A♂}+2Cov_{A♀♂}$$

and the corresponding total heritability as

(11) $$T^{2}=V_{A\;total}/V_{P}.$$

Although our approach accounts for the effects of female and male pair members, we note that the indirect genetic effects relevant for an individual's use of social cues for breeding site choice may not be limited to within the breeding pairs but may also include, for example, the other conspecifics (pairs) breeding in the neighborhood. If such indirect genetic effects exist, our estimates of “total” additive genetic variance and heritability are somewhat biased low compared to the real total additive genetic variance and heritability (cf. Bijma et al. 2007a,b; Bergsma et al. 2008; Bijma 2011). Future work in the joint phenotype – indirect genetic effects context could consider both the effects of individuals sharing the phenotype and the effects of their social associates (not sharing the phenotype), but we do not pursue that here.

Statistical analyses were performed within the Bayesian statistical framework using R version 3.5.1 (R Development Core Team 2018). Repeatability and quantitative genetic models with Gaussian distributions for the response variables were fitted using the function “MCMCglmm” (R package MCMCglmm; Hadfield 2010). We used inverse Wishart prior (V = 1, nu = 1) for the residual variance and parameter expanded priors (V = 1, nu = 1, alpha.mu = 0, alpha.V = 1000; Gelman 2006) for the other variance components. Analyses using alternative prior specifications resulted in qualitatively identical variance component estimates (see Supporting Information, Section E). For each model, we run three MCMC chains for 2,550,000 iterations with burn‐in of 50,000 and thinning interval of 500, resulting in 5000 stored parameter estimates from the posterior distribution per chain. Visual evaluation of trace plots and Gelman‐Rubin diagnostics (all potential scale reduction factors <1.05) suggested adequate convergence of the MCMC chains in all analyses. Autocorrelations of the stored parameter estimates were below 0.1 and the effective sample sizes were at least 13,735 (details in the tables in Supporting Information, Section G). We report the posterior medians and the 95% highest posterior density credibility intervals of the parameter estimates (function “HPDinterval”; Plummer et al. 2006). See Supporting Information, Section F, for R syntax of the statistical models.

## Results

### REPEATABILITIES IN THE USE OF SOCIAL CUES FOR BREEDING SITE CHOICE

**Figure 1:**
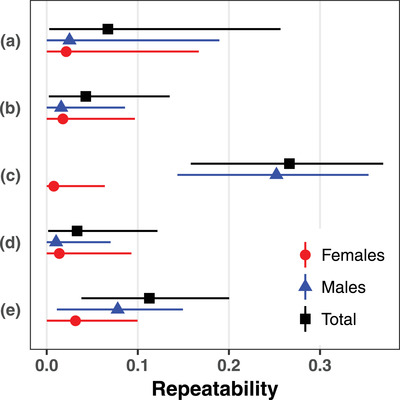
Estimates of female (red circles), male (blue triangles), and total (black squares) repeatabilities (median ± 95% CI) in the use of (A) conspecific abundance in the current year, (B) conspecific abundance in the previous year, (C) conspecific success in the previous year, (D) great tit abundance in the current year, and (E) great tit abundance in the previous year as social cues for breeding site choice by collared flycatchers.

The total repeatabilities in the use of con‐ and heterospecific social cues for breeding site choice by collared flycatchers were relatively low. Visual inspection of the posterior distributions suggested support for repeatability for two out of the five social cues considered: (i) previous year conspecific success (median [95% CI]: 0.27 [0.16 to 0.37]) and (ii) previous year great tit abundance (0.11 [0.038 to 0.20]; Table 2; Fig. 1). The median total repeatabilities for the three other social cues ranged from 0.033 to 0.067, but their lower 95% CI limits were very close to zero (Table 2; Fig. 1). When separating sexes, the median repeatabilities for females ranged from 0.0078 to 0.032 and for males from 0.010 to 0.25 (Table 2; Fig. 1). The estimates of the variance components and derived metrics for the five social cues are reported in Table 2, and the fixed effects estimates in Table S4 (Supporting Information, Section G). The repeatability estimates remained similar when no fixed effect but the intercept was included in the models (Table S5 in Supporting Information, Section G).

**Table 2:** Median estimates and their 95% CIs (in parentheses) for the variance components in the univariate GLMMs estimating repeatability for the use of five social cues for breeding site choice in collared flycatcher. Estimates are conditional on the fixed effects. *V*_PI♀_ and *V*_PI♂_ are the female and male permanent individual variances, *V*_PATCH_ is the spatial variance across forest patches, *V*_BOX_ is the variance between nest boxes, and *V*_R_ is the residual variance. Also the derived metrics total phenotypic variance *V*_P_, total permanent individual variance *V*_PI total_, and female *R*_♀_, male *R*_♂_, and total *R*_total_ repeatabilities are reported

	Conspecific abundance in the current year	Conspecific abundance in the previous year	Conspecific success in the previous year	Great tit abundance in the current year	Great tit abundance in the previous year
Variance components					
*V*_PI♀_	0.016	0.016	0.0076	0.0090	0.026
	(4.3 × 10^−5^ to 0.13)	(4.8 × 10^−5^ to 0.090)	(1.8 × 10^−5^ to 0.063)	(2.4 × 10^−5^ to 0.061)	(1.3 × 10^−4^ to 0.082)
*V*_PI♂_	0.019	0.015	0.24	0.0067	0.064
	(4.2 × 10^−5^ to 0.14)	(3.9 × 10^−5^ to 0.080)	(0.14 to 0.36)	(1.4 × 10^−5^ to 0.046)	(0.0093 to 0.12)
*V*_PATCH_	0.058	0.0012	0.015	0.0034	0.072
	(0.024 to 0.16)	(2.5 × 10^−6^ to 0.014)	(0.0029 to 0.057)	(1.1 × 10^−5^ to 0.027)	(0.031 to 0.20)
*V*_BOX_	0.018	0.045	0.012	0.068	0.41
	(4.5 × 10^−5^ to 0.11)	(6.8 × 10^−4^ to 0.11)	(3.2 × 10^−5^ to 0.080)	(0.012 to 0.13)	(0.33 to 0.49)
*V*_R_	0.59	0.82	0.67	0.54	0.24
	(0.45 to 0.68)	(0.72 to 0.92)	(0.58 to 0.78)	(0.47 to 0.62)	(0.19 to 0.29)
Derived metrics					
*V*_P_	0.74	0.92	0.97	0.64	0.82
	(0.67 to 0.85)	(0.86 to 0.99)	(0.90 to 1.1)	(0.60 to 0.70)	(0.74 to 0.96)
*V*_PI total_	0.050	0.039	0.26	0.022	0.093
	(0.0021 to 0.19)	(0.0022 to 0.13)	(0.15 to 0.37)	(9.9 × 10^−4^ to 0.080)	(0.032 to 0.16)
*R*_♀_	0.021	0.018	0.0078	0.014	0.032
	(5.8 × 10^−5^ to 0.17)	(5.3 × 10^−5^ to 0.097)	(1.9 × 10^−5^ to 0.064)	(3.6 × 10^−5^ to 0.093)	(1.6 × 10^−4^ to 0.10)
*R*_♂_	0.025	0.016	0.25	0.010	0.078
	(5.7 × 10^−5^ to 0.19)	(4.3 × 10^−5^ to 0.086)	(0.14 to 0.35)	(2.2 × 10^−5^ to 0.070)	(0.011 to 0.15)
*R*_total_	0.067	0.043	0.27	0.033	0.11
	(0.0028 to 0.26)	(0.0023 to 0.13)	(0.16 to 0.37)	(0.0015 to 0.12)	(0.038 to 0.20)

### ADDITIVE GENETIC (CO)VARIANCES AND HERITABILITY ESTIMATES OF THE USE OF SOCIAL CUES

**Figure 2:**
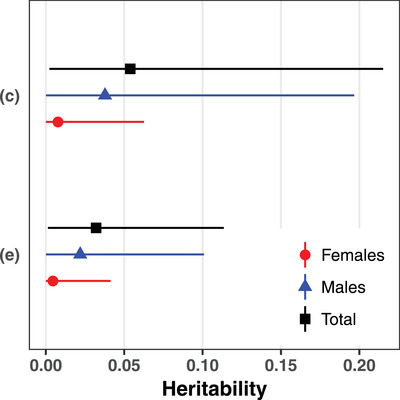
Estimates of female (red circles), male (blue triangles), and total (black squares) heritabilities (median ± 95% CI) in the use of (C) conspecific success in the previous year and (E) great tit abundance in the previous year as social cues for breeding site choice by collared flycatchers.

The quantitative genetic models including cross‐sex additive genetic covariance estimated the covariance to be not significantly different from zero for both repeatable social cues: (i) –3.0 × 10^−5^ [–0.031 to 0.026] (median [95% CI]) for conspecific success in the previous year and (ii) –4.6 × 10^−5^ [–0.016 to 0.012] for great tit abundance in the previous year (Table S6 in Supporting Information, Section G). Thus, we estimated heritabilities using models excluding the cross‐sex additive genetic covariance. These models suggested low heritabilities (Table 3; Fig. 2) and additive genetic variances (Table 3; Fig. S3 in Supporting Information, Section G) for the use of both social cues for breeding site choice. The estimates of the variance components and derived metrics are reported in Table 3, and the fixed effects estimates in Table S7 (Supporting Information, Section G). All estimates remained similar when no fixed effect but the intercept was included in the quantitative genetic models (Table S9 in Supporting Information, Section G).

**Table 3:** Median estimates and their 95% CIs (in parentheses) for the variance components in the univariate GLMMs estimating additive genetic variance and heritability for the use of two social cues for breeding site choice in collared flycatcher. Estimates are conditional on the fixed effects. *V*_A♀_ and *V*_A♂_ are the female and male additive genetic variances, *V*_DOM♀_ and *V*_DOM♂_ are the female and male dominance genetic variances, *V*_PI♀_ and *V*_PI♂_ are the female and male permanent individual variances, *V*_PATCH_ is the spatial variance across forest patches, *V*_BOX_ is the variance between nest boxes, and *V*_R_ is the residual variance. Also the derived metrics total additive genetic variance *V*_A total_, total phenotypic variance *V*_P_, and female $h_{♀\;}^{2}$, male $h_{♂}^{2}$, and total heritabilities *T* ^2^ are reported

	Conspecific success in the previous year		Great tit abundance in the previous year	
Variance components				
*V*_A♀_	0.0076	(1.6 × 10^−5^ to 0.062)	0.0037	(7.8 × 10^−6^ to 0.034)
*V*_A♂_	0.037	(9.9 × 10^−5^ to 0.19)	0.018	(5.7 × 10^−5^ to 0.082)
*V*_DOM♀_	0.0064	(1.3 × 10^−5^ to 0.055)	0.010	(2.6 × 10^−5^ to 0.060)
*V*_DOM♂_	0.093	(3.5 × 10^−4^ to 0.28)	0.018	(4.5 × 10^−5^ to 0.089)
*V*_PI♀_	0.0061	(1.4 × 10^−5^ to 0.053)	0.012	(2.9 × 10^−5^ to 0.066)
*V*_PI♂_	0.067	(1.6 × 10^−4^ to 0.27)	0.018	(4.1 × 10^−5^ to 0.089)
*V*_PATCH_	0.015	(0.0028 to 0.058)	0.072	(0.031 to 0.21)
*V*_BOX_	0.012	(2.9 × 10^−5^ to 0.078)	0.40	(0.32 to 0.48)
*V*_R_	0.66	(0.56 to 0.76)	0.22	(0.18 to 0.27)
Derived metrics				
*V*_P_	0.98	(0.90 to 1.1)	0.82	(0.74 to 0.96)
*V*_A total_	0.052	(0.0022 to 0.21)	0.026	(0.0011 to 0.092)
$h_{♀\;}^{2}$	0.0077	(1.6 × 10^−5^ to 0.063)	0.0045	(9.4 × 10^−6^ to 0.041)
$h_{♂\;}^{2}$	0.038	(9.9 × 10^−5^ to 0.20)	0.022	(7.1 × 10^−5^ to 0.10)
*T* ^2^	0.054	(0.0023 to 0.22)	0.032	(0.0013 to 0.11)

## Discussion

We assessed the individual repeatability, additive genetic variance, and heritability of the use of con‐ and heterospecific social cues for breeding site choice in a population of wild collared flycatchers. Social information from both con‐ and heterospecifics was found to influence breeding site choice of collared flycatchers (Figure S2 in Supporting Information, Section C), confirming previous results based on different datasets (Doligez et al. 1999, 2002; Seppänen and Forsman 2007; Kivelä et al. 2014; Jaakkonen et al. 2015; Morinay et al. 2020a,b). Yet, individual repeatabilities of the use of social cues were low both at the level of the total population (range: 0.033–0.27) and within sexes (0.0078–0.032 for females and 0.010–0.25 for males). These repeatabilities are lower than those reported for most behavioral traits in animals (see review in Bell et al. 2009), including the repeatability of habitat choice (0.60 on average; Bell et al. 2009), but are in line with the low estimates obtained for copying heterospecific nest site characteristics in this population (Morinay et al. 2018). Here, the posterior distributions of total repeatabilities were away from zero for the use of only two of the social cues, namely, conspecific success and great tit abundance in the previous year, for breeding site choice; yet the median estimates were low (0.11–0.27). Because repeatability indicates the potential maximal heritability level (Nagakawa and Schielzeth 2010), we not surprisingly found that additive genetic variances and heritabilities (female, male, and total heritability) were also low for the use of both conspecific success (medians 0.0077–0.054) and great tit abundance in the previous year (medians 0.0045–0.032) for breeding site choice. The heritability estimates of the traits investigated here were considerably lower than the average heritability level of behavioral traits, estimated to 0.235 (reviewed by Dochtermann et al. 2019). Given that both female and male additive genetic variances were low, we found no evidence for nonzero additive genetic covariance between females and males. Thus, cross‐sex genetic correlations are unlikely to affect the evolutionary dynamics of the joint breeding site choice in relation to social cues in this population.

A recent study in the same collared flycatcher population examined the genetic determinism of social information use in terms of copying nest site characteristics of heterospecific tits and also found low additive genetic variation and no cross‐sex additive genetic correlation for this behavior (Morinay et al. 2018). These previous results together with ours show a different picture from those of laboratory studies in *Drosophila* fruit flies and in a colonial passerine bird, which revealed a strong genetic basis for the variation in social information use and social environment choice based on conspecifics (Brown and Brown 2000; Saltz 2011; Foucaud et al. 2013; Philippe et al. 2016; Geiger and Saltz 2020). Our results in a solitarily breeding species suggest the opposite, that is, a weak genetic basis for the variation in this behavior. However, cross‐sex genetic correlation for social environment choice seems to be absent also in *Drosophila*, despite existing genetic variation in both sexes (Geiger and Saltz 2020). Previous work on other social traits suggests varying levels of genetic determinism across traits and species (Fairbanks et al. 2004; Charmantier et al. 2007; Lea et al. 2010; Brent et al. 2013; Weiss and Foerster 2013; Blomquist and Brent 2014; Araya‐Ajoy and Dingemanse 2017), although part of the variation in the estimates may be due to methodological reasons (e.g., model structure; Wilson 2008). For example, the level of heritability of aggressive behavior in mammals varied from 0.02 (±0.05 SE) in yellow‐bellied marmots *Marmota flaviventris* (Lea et al. 2010) up to 0.66 (±0.28 SE) in rhesus macaques *Macaca mulatta* (Brent et al. 2013). Whether similar variation in genetic determinism of social information use for breeding decisions is common remains an open question until more estimates from other natural populations become available.

Heterospecifics influence the competitive environment and information transmission patterns (e.g., Seppänen et al. 2007; Goodale et al. 2010; Farine et al. 2015) and therefore constitute an important component of an individual's social environment (Seppänen et al. 2007; Goodale et al. 2010). Thus, accounting for interspecific interactions in the choice of social environment is important for understanding the effects of social environment choice comprehensively. The lack of genetic variation in the use of heterospecific social cues observed here, however, suggests that the potential for genetically driven variation in the associations and interactions with heterospecific competitors during breeding is low, at least in this population (see also Morinay et al. 2018).

The low additive genetic variances in the use of social cues for breeding site choice may be the result of consistent and strong directional selection (e.g., Gustafsson 1986; Mousseau and Roff 1987; Lynch and Walsh 1998; Merilä and Sheldon 1999; Gustafsson and Qvarnström 2006) favoring the use of social cues in a specific way, for example, attraction to conspecifics or heterospecifics instead of avoidance or neutral behavior. Social information use for breeding site choice and in particular the attraction to previously successful conspecifics or ecologically similar heterospecifics can enhance the information user's own reproductive success (Schjørring et al. 1999; Forsman et al. 2002; Seppänen et al. 2005; Pärt et al. 2011). Thus, breeding close to con‐ or heterospecifics could be under positive directional selection, which could thereby decrease additive genetic variance in the use of these information sources. There may also be disadvantages of breeding too close to conspecifics or heterospecifics (e.g., increased competition, aggressiveness, and harassment), hence leading to an optimum distance to or density of neighbors (Seppänen et al. 2007). If this optimum was reached, stabilizing selection may also have contributed to reduce the additive genetic variance.

The low repeatabilities and heritabilities suggest that the phenotypic variation in the use of social information for breeding site choice in this population may result from variable environmental conditions, rather than additive genetic or other permanent individual effects. If environmental variation is associated with the fitness consequences of the use of social information, such phenotypic variation could be expected. Empirical studies showing fitness benefits of the use of social information in breeding site choice (Schjørring et al. 1999; Forsman et al. 2002; Seppänen et al. 2005; Pärt et al. 2011) have not considered environmentally mediated variation, but theoretical work suggests that the fitness benefits of, for example, conspecific and heterospecific attraction should decrease at high breeding densities (Doligez et al. 2003; Mönkkönen et al. 2004). If breeding densities vary in time and/or space, so could the fitness benefits of attraction. Such variation in fitness effects has been described in the context of mate choice in our study population (Robinson et al. 2012; see also Teerikorpi et al. 2018). If fitness prospects vary in time or space such that attraction to conspecifics and/or heterospecifics is favored at low to intermediate breeding densities and avoidance is favored at high densities, plasticity in the use of these social cues would be adaptive. Phenotypic plasticity is common in animal behavior (Nussey et al. 2007; Dingemanse et al. 2010; Brommer 2013) and may itself be heritable and under selection (Nussey et al. 2005; Araya‐Ajoy and Dingemanse 2017; but see Brommer et al. 2005 and van Heerwaarden and Sgro 2017 for contrasting results). Thus, studying the plasticity of the use of social information and its genetic basis could also help achieve a better understanding of the inheritance mechanisms of the use of social information.

The evolutionary potential of a trait in natural populations is traditionally expected to be directly related to the amount of additive genetic variance underlying the variation in the trait (e.g., Fisher 1930; Houle 1992). Negligible additive genetic variance would thus suggest that the evolutionary potential of the use of social information for breeding site choice in this flycatcher population is very limited. However, genetic parameters obtained in a single study, over a certain (limited) time frame and environmental conditions, may not provide a representative picture of the trait's evolutionary potential in a population. For example, several studies have shown that the amount of (observable) additive genetic variance can vary with environmental conditions (Hoffmann and Merilä 1999; Charmantier and Garant 2005; Wilson et al. 2006; Brommer et al. 2008). The population may exhibit “cryptic” genetic variation that is not reflected in the phenotypic variation in the current conditions, but becomes observable when environmental conditions change (Hoffmann and Merilä 1999; Charmantier and Garant 2005; Wilson et al. 2006; Brommer et al. 2008). Furthermore, indirect genetic effects stemming from the breeding site choice of neighboring conspecifics may contribute to a higher total additive genetic variance and heritability of the use of social cues than estimated here (cf. Moore et al. 1997; Wolf et al. 1998; Wolf 2003; Bijma et al. 2007a; Bijma 2011). Finally, nongenetic inheritance mechanisms, for example, epigenetic, environmental, and social (cultural) inheritance, may also contribute to the evolutionary potential of traits (Jablonka and Lamb 2005; Bonduriansky and Day 2009; Danchin et al. 2011; Danchin 2013). To what extent the nongenetic inheritance mechanisms play a role in the evolutionary potential of social information use remains to be explored.

In conclusion, we found low additive genetic variation underlying phenotypic variation in the use of conspecific and heterospecific social cues for breeding site choice in a wild collared flycatcher population. Our work contrasts with previous results in other taxa suggesting differences in the amount of genetic variation in the use of social information among traits and/or species. More work on the genetics of the use of social information in general is needed. Future work should also address within‐individual plasticity in the use of social information, due to learning, for example, which may be more adaptive in changing environmental conditions than genetically fixed strategies, even though the ability to learn could also be genetically determined and under positive selection.

## AUTHOR CONTRIBUTIONS

JF, SK, JT, JM, and BD designed the study. BD, JF, JT, JM, SK, and many field assistants carried out the field work. JT, SK, and JM prepared and JT analyzed the data. JT drafted the manuscript. All authors provided critical input to the manuscript.

## CONFLICT OF INTEREST

The authors declare no conflict of interest.

## DATA ARCHIVING

The data used in this article have been uploaded to Dryad (https://doi.org/10.5061/dryad.pzgmsbchv).

Associate Editor: K. McGuigan

Handling Editor: D. W. Hall

## Supporting information


**Figure S1**. Map of the study area. Dots illustrate the locations of nest boxes and different colors depict the 15 forest patches. Background map is the OpenStreetMap.
**Table S1**. Characteristics of the 15 forest patches included in the study.
**Figure S2**. Summary of the use of (a) conspecific abundance in the current year, (b) conspecific abundance in the previous year, (c) conspecific success in the previous year , (d) great tit abundance in the current year, and (e) great tit abundance in the previous year as social cues for breeding site choice by collared flycatchers.
**Table S2**. Comparison of the variance component estimates in the repeatability models with varying prior specifications.
**Table S3**. Comparison of the variance component estimates in the quantitative genetic models (full models including the cross‐sex additive genetic covariance) with varying prior specifications.
**Table S4**. Parameter estimates (posterior medians) and their 95% credibility intervals in the univariate GLMM estimating repeatability for the use of five social cues for breeding site choice in collared flycatcher
**Table S5**. Parameter estimates (posterior medians) and their 95% credibility intervals in the univariate GLMM estimating repeatability for the use of five social cues for breeding site choice in collared flycatcher
**Figure S3**. Estimates of female (red circles), male (blue triangles) and total (black squares) additive genetic variances (median ± 95% CI) in the use of (c) conspecific success in the previous year and (e) great tit abundance in the previous year as social cues for breeding site choice by collared flycatchers, based on the models with the full fixed effects structure, but excluding the cross‐sex additive genetic covariance (see Table S7).
**Table S6**. Parameter estimates (posterior medians) and their 95% credibility intervals in the univariate GLMM estimating additive genetic variance and heritability for the use of two social cues for breeding site choice in collared flycatcher.
**Table S7**. Parameter estimates (posterior medians) and their 95% credibility intervals in the univariate GLMM estimating additive genetic variance and heritability for the use of two social cues for breeding site choice in collared flycatcher.
**Table S8**. Parameter estimates (posterior medians) and their 95% credibility intervals in the univariate GLMM estimating additive genetic variance and heritability for the use of two social cues for breeding site choice in collared flycatcher.
**Table S9**. Parameter estimates (posterior medians) and their 95% credibility intervals in the univariate GLMM estimating additive genetic variance and heritability for the use of two social cues for breeding site choice in collared flycatcher.
**Table S10**. Comparison of the repeatability estimates derived from the repeatability and the quantitative genetic models.
