# Supplementary material for: Quantitative genetics of the use of conspecific and heterospecific social cues for breeding site choice
Source: Evolution. 2020 Aug 13;74(10):2332–47. doi: 10.1111/evo.14071 (PMC7589285; doi:10.1111/evo.14071)
Supplement: Supplementary file 1 — Figure S1. Map of the study area. Dots illustrate the locations of nest boxes and different colors depict the 15 forest patches. Background map is the OpenStreetMap. Table S1. Characteristics of the 15 forest patches included in the study. Figure S2. Summary of the use of (a) conspecific abundance in the current year, (b) conspecific abundance in the previous year, (c) conspecific success in the previous year , (d) great tit abundance in the current year, and (e) great tit abundance in the previous year as social cues for breeding site choice by collared flycatchers. Table S2. Comparison of the variance component estimates in the repeatability models with varying prior specifications. Table S3. Comparison of the variance component estimates in the quantitative genetic models (full models including the cross‐sex additive genetic covariance) with varying prior specifications. Table S4. Parameter estimates (posterior medians) and their 95% credibility intervals in the univariate GLMM estimating repeatability for the use of five social cues for breeding site choice in collared flycatcher Table S5. Parameter estimates (posterior medians) and their 95% credibility intervals in the univariate GLMM estimating repeatability for the use of five social cues for breeding site choice in collared flycatcher Figure S3. Estimates of female (red circles), male (blue triangles) and total (black squares) additive genetic variances (median ± 95% CI) in the use of (c) conspecific success in the previous year and (e) great tit abundance in the previous year as social cues for breeding site choice by collared flycatchers, based on the models with the full fixed effects structure, but excluding the cross‐sex additive genetic covariance (see Table S7). Table S6. Parameter estimates (posterior medians) and their 95% credibility intervals in the univariate GLMM estimating additive genetic variance and heritability for the use of two social cues for breeding site choice in collared flycatcher. [file EVO-74-2332-s001.pdf]

Supporting Information

Quantitative genetics of the use of conspecific and heterospecific social cues for breeding site choice

Section A. Description of the study area

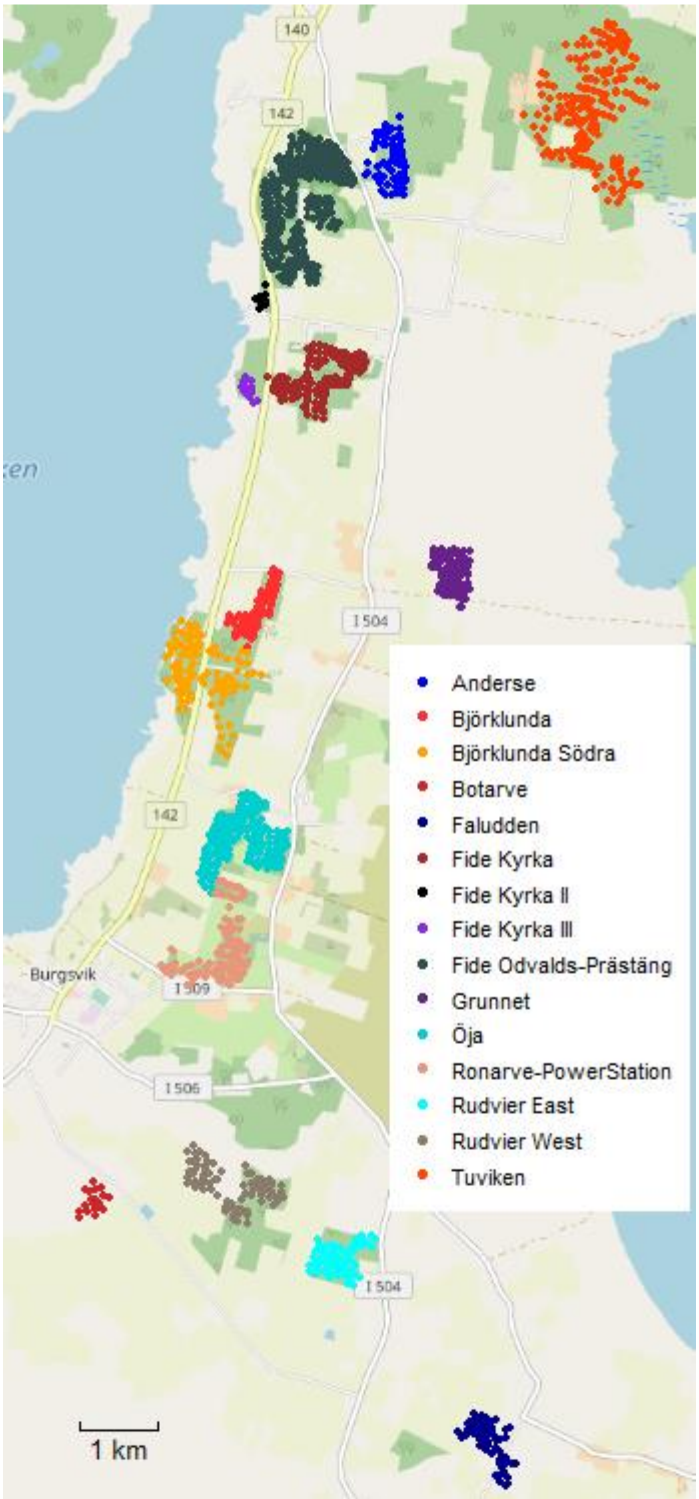

Figure S1. Map of the study area. Dots illustrate the locations of nest boxes and different colors depict the fifteen forest patches. Background map is the OpenStreetMap (© OpenStreetMap contributors).

Table S1. Characteristics of the 15 forest patches included in the study. Average minimum distance between nest boxes is the average distance between each nest box and its closest neighboring box.

Patch name	Patch area (ha)	Number of nest boxes	Average minimum distance between nest boxes (m)	Maximum distance between nest boxes (m)
Anderse	11.5	61	33	549
Björklunda	9.3	64	25	575
Björklunda Södra	21.6	104	37	1000
Botarve	4.2	18	41	268
Faludden	10.1	65	27	543
Fide Kyrka	18.9	137	24	702
Fide Kyrka II	1.7	10	32	168
Fide Kyrka III	2.3	13	27	186
Fide Odvalds-Prästäng	40.8	336	21	1053
Grunnet	10.6	76	25	449
Öja	24.2	164	27	753
Ronarve-PowerStation	17.6	93	30	787
Rudvier East	11.0	65	27	523
Rudvier West	15.4	78	33	736
Tuviken	39.5	197	34	1260

Section B. Derivation of the response variables

Derivation of the response variables describing the use of social cues in breeding site choice was identical to that in Kivelä et al. (2014). Because we were unable to determine the exact dates of breeding site choice (initiation of nest building), we assumed that it took place five days before egg laying started, which is approximately the average time interval observed recently (pers. obs.). Kivelä et al. (2014) tested for the impact of this assumption by repeating analyses using two other time intervals between nest initiation and egg laying (two and eight days); the results were robust to variation in the time interval used.

Response variables describing breeding site choice in relation to current and previous year abundance of conspecifics and great tits were derived by comparing the effective number of neighbors of the species considered around the nest box chosen by the focal collared flycatcher pair to the average effective number of neighbors around all available boxes (including the chosen one) in the forest patch on the same day as the focal pair was assumed to have made its breeding site choice. This was done by calculating the difference variable D_O:

$$D_O(x, \alpha, i, k, t, T - \Delta) = O(x, \alpha, i, k, t, T - \Delta) - E[O(x, \alpha, k, t, T - \Delta)] \quad \text{(S1)}$$

where $O(x, \alpha, i, k, t, T - \Delta)$ is the effective number of neighbors (number of neighbors weighed by inverse distance) of species x (x = collared flycatcher, great tit) around the chosen nest box i in the forest patch k on day t in year $T - \Delta$ (T = 2005,..., 2010). $\Delta = 0$ refers to the situation on the day of breeding site choice (day t in year T) and $\Delta = 1$ to the situation at the end of the previous breeding season (year $T - 1$). α (in meters) determines the spatial scale over which the number of neighbors is calculated (see below). $O(x, \alpha, i, k, t, T - \Delta)$ was calculated as

$$O(x, \alpha, i, k, t, T - \Delta) = \sum_{j=1}^{n_{k(T-\Delta)}} e^{\frac{-d_{ij}}{\alpha}} o(x, j, k, t, T - \Delta) \quad \text{(S2)}$$

where d_{ij} is the distance (in meters) between the focal nest box i and a nest box j (j = 1,..., $n_{k(T-\Delta)}$, where $n_{k(T-\Delta)}$ is the number of nest boxes in forest patch k in year ($T - \Delta$). The weighing coefficient $e^{-(d_{ij}/\alpha)}$ decreases exponentially with increasing distance between nest boxes i and j, d_{ij}, the rate of decrease increasing with decreasing values of α. $o(x, j, k, t, T - \Delta) = 1$ if the nest box j was occupied by species x (x = collared flycatcher, great tit; on day t if $\Delta = 0$) in the breeding season $T - \Delta$, and otherwise $o(x, j, k, t, T - \Delta) = 0$.

$E[O(x, \alpha, k, t, T - \Delta)]$ is the expected number of species x neighbors in year $T - \Delta$ calculated by averaging the effective number of neighbors in year $T - \Delta$ of all nest boxes available (empty) in the forest patch k on day t in year T. To take into account potential intrinsic preference of collared flycatchers for certain breeding

sites, $E[O(x, \alpha, k, t, T-\Delta)]$ was calculated as a weighted average, each nest box being weighted by its probability of occupancy by collared flycatchers during the period 1990–2000. We used the period 1990-2000 to avoid having the same individuals in both the data set used for estimating the general preference of collared flycatchers for certain nest boxes and for the data set used in the repeatability and heritability analyses. In other words, we wanted an independent sample of individuals to estimate the preference for certain nest boxes. Years earlier than 1990 were not included, because they may not have reflected habitat quality at the sites later on, here between 2005 and 2010, due to forestry management and forest succession

To define breeding site choice in relation to previous year breeding success of conspecifics and great tits, we derived the effective average number of fledglings $F(x, \alpha, i, k, t, T-1)$ in the neighborhood of the focal nest box i produced by species x in the previous breeding season $T-1$ as

$$F(x, \alpha, i, k, t, T-1) = \frac{\sum_{j=1}^{n_{k(T-1)}} e^{\frac{-d_{ij}}{\alpha}} f(x,j,k,t,T-1)}{\sum_{j=1}^{n_{k(T-1)}} e^{\frac{-d_{ij}}{\alpha}} o(x,j,k,t,T-1)} \quad \text{(S3)}$$

where $f(x,j,k,t,T-1)$ is the number of fledglings produced by species x in the nest box j of the forest patch k. The difference variable D_F was calculated as

$$D_F(x, \alpha, i, k, t, T-1) = F(x, \alpha, i, k, t, T-1) - E[F(x, \alpha, k, t, T-1)] \quad \text{(S4)}$$

where $E[F(x, \alpha, k, t, T-1)]$ is the expected average number of fledglings produced by species x in the previous year in the neighborhood of the available nest boxes in forest patch k on day t in year T. As for $E[O(x, \alpha, k, t, T-\Delta)]$, we calculated $E[F(x, \alpha, k, t, T-1)]$ as a weighted average incorporating collared flycatcher occupancy probabilities in 1990–2000.

The four $D_O(x, \alpha, i, k, t, T-\Delta)$ and two $D_F(x, \alpha, i, k, t, T-1)$ variables were normalized (see main text) for analyses. These variables were then used as the response variables in the quantitative genetic (animal model) analyses.

Section C. Summary of the use of the social cues

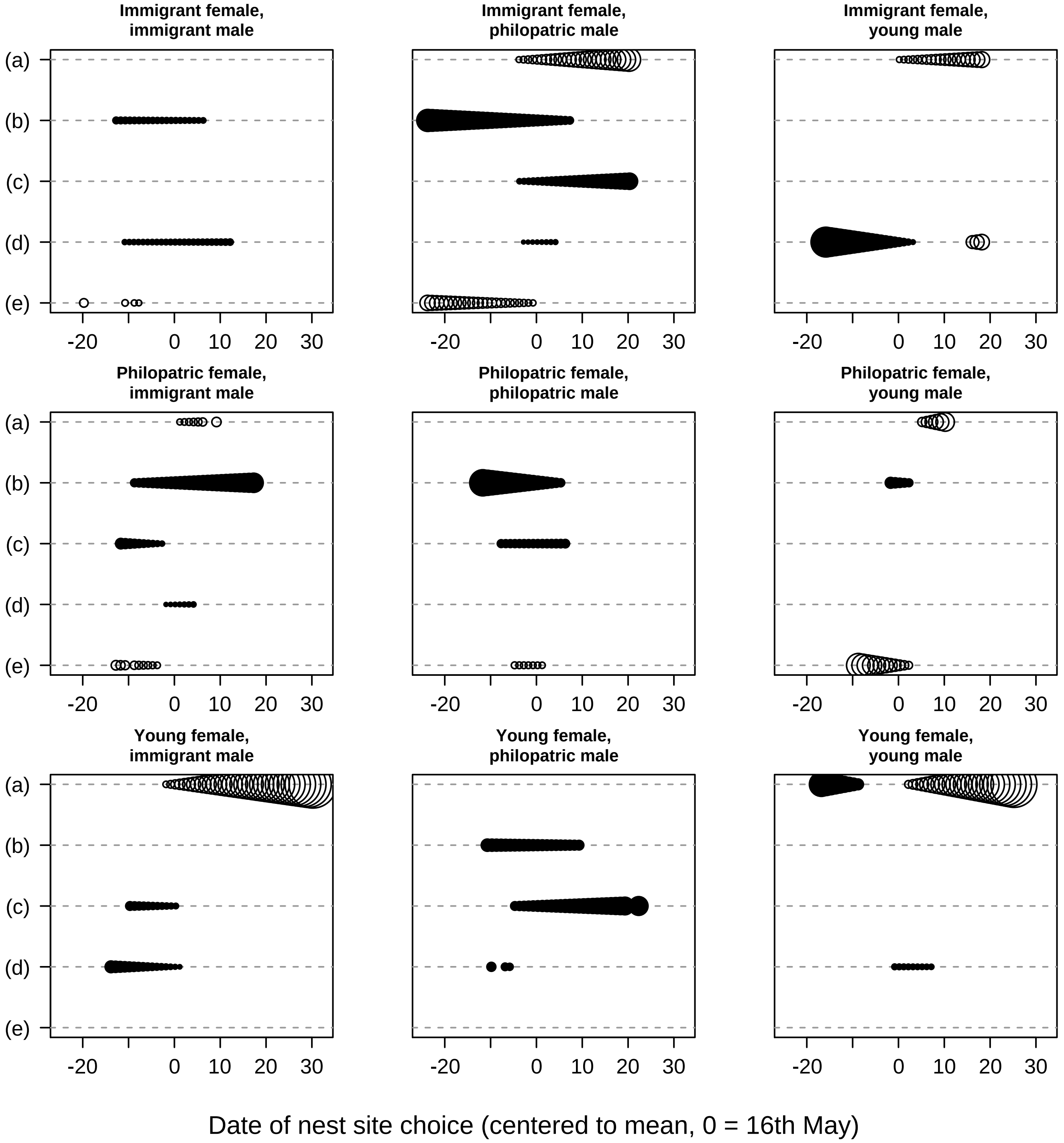

Figure S2. Summary of the use of (a) conspecific abundance in the current year, (b) conspecific abundance in the previous year, (c) conspecific success in the previous year , (d) great tit abundance in the current year and (e) great tit abundance in the previous year as social cues for breeding site choice by collared flycatchers. Each panel represents one of the nine pair types (female-male "Status" combinations) and illustrates the part of the settlement period when the social cue is used. Closed symbols denote positive response variable values (i.e., attraction) and open symbols negative response variable values (i.e., avoidance). Increasing relative symbol size illustrates increasing deviation (absolute value) from zero (i.e., stronger attraction or avoidance). Only cases where the 95% CIs of the fitted regression line excluded zero are shown.

The predicted estimates for the use of the social cues for breeding site choice for the different collared flycatcher pair combinations (combinations of the female and male “Status” variables), based on the *repeatability models* (see Tables S4), were similar to those found in Kivelä et al. (2014) (Figure S2). For all five social cues, most of the pair combinations showed significant attraction or avoidance, but varied in the timing of the use of the cues. Breeding sites with high current conspecific abundance were avoided by late settling pairs consisting of at least one young or immigrant pair member (Figure S2 a). Pairs of young individuals also showed attraction to conspecifics early in the season. In contrast, high conspecific abundance and breeding success in the previous year elicited attraction, especially in pairs including philopatric individuals (Figure S2 b, c). Pairs including immigrant or young individuals were in most cases attracted by relatively high current great tit abundance (Figure S2 d), whereas breeding sites with high great tit abundance in the previous year were avoided early in the breeding season (Figure S2 e).

Section D. Statistics of the pruned pedigree

records	3557
maternities	1278
paternities	1192
full sibs	222
maternal sibs	454
maternal half sibs	232
paternal sibs	428
paternal half sibs	206
maternal grandmothers	455
maternal grandfathers	436
paternal grandmothers	442
paternal grandfathers	391
maximum pedigree depth	14
founders	2264
mean maternal sibship size	1.327
mean paternal sibship size	1.332
non-zero F	6
F > 0.125	1
mean pairwise relatedness	0.00037
proportion of pairwise relatedness values >=0.125	0.00114
proportion of pairwise relatedness values >=0.25	0.00081
proportion of pairwise relatedness values >=0.5	0.00043

Section E. Effects of prior choice on the variance component estimates

For both the repeatability models and quantitative genetic models, the original prior specification included an inverse Wishart prior for the residual variance and parameter expanded priors for the other variance components. For the repeatability models, the R syntax for the prior specification was:

```
prior <- list(R=list(V=1 ,nu=1),
G=list(G1= list(V=1, nu=1, alpha.mu=0, alpha.V=1000),
G2=list(V=1, nu=1, alpha.mu=0, alpha.V=1000),
G3=list(V=1, nu=1, alpha.mu=0, alpha.V=1000),
G4=list(V=1, nu=1, alpha.mu=0, alpha.V=1000)))
```

where *R* refers to residual variance and *G1-G4* to the female and male permanent individual variances, between-patch and between-nest box variances, respectively. For the quantitative genetic models, the R syntax for the prior specification was:

```
prior <- list(R=list(V=1 ,nu=1),
G=list(G1=list(V=diag(2), nu=2, alpha.mu=c(0,0), alpha.V=diag(2)*1000),
G2=list(V=1, nu=1, alpha.mu=0, alpha.V=1000),
...,
G7=list(V=1, nu=1, alpha.mu=0, alpha.V=1000)))
```

where *R* refers to residual variance, *G1* to additive genetic (co)variances and *G2-G7* to the other variance components.

We evaluated the effect of prior choice on the variance component estimates by varying *V* and *nu*. Following Wilson et al. (2010, Supplementary File 5: Tutorial for MCMCglmm version), alternative *V* parameters were defined in relation to the total phenotypic variance such that in each alternative prior specification, one variance component had *V* equal to 95% of the total phenotypic variance (i.e., V=0.95*Vp), while for the other variance components we used V=(0.05/4)*Vp (repeatability models) or V=(0.05/8)*Vp (quantitative genetic models). Each variance component was associated with V=0.95*Vp at a time, resulting in five different alternative prior specifications for the repeatability models and nine different alternative prior specifications for the quantitative genetic models. We also increased *nu*=1 in the original prior to *nu*=10 in the alternative priors to impose a more informative (more peaked) prior distributions on the variance components. The R syntax for the resulting alternative prior was (for example, the prior with V=0.95*Vp for female additive genetic variance in the quantitative genetic models):

```
prior_AF95 <- list(R=list(V=(0.05/8)*Vp, nu=10),
G=list(G1=list(V=diag(c(0.95*Vp,(0.05/8)*Vp)), nu=20, alpha.mu=c(0,0), alpha.V=diag(2)*1000),
G2=list(V=(0.05/8)*Vp, nu=10, alpha.mu=0, alpha.V=1000),
...,
G7=list(V=(0.05/8)*Vp, nu=10, alpha.mu=0, alpha.V=1000)))
```

We tested the effect of prior specification using the maximum models fitted in the original analyses (i.e. full fixed effects structure, and including the cross-sex additive genetic covariance in the quantitative genetic models). Estimates of the variance components were qualitatively and quantitatively similar irrespective of the prior specification (Tables S2 and S3).

Table S2. Comparison of the variance component estimates in the repeatability models with varying prior specifications. Median estimates using the original prior and the range of median estimates (in parentheses; with the accuracy of two significant digits) using the five alternative priors are presented for each variable.

**Response variable**	$V_{\mathrm{PI}♀}$	$V_{\mathrm{PI}♂}$	V_{PATCH}	V_{BOX}	V_{R}
Conspecific abundance, current year	0.016 (0.015-0.028)	0.019 (0.018-0.036)	0.058 (0.057-0.059)	0.018 (0.017-0.028)	0.59 (0.55-0.60)
Conspecific abundance, previous year	0.016 (0.016-0.021)	0.015 (0.015-0.017)	0.0012 (0.0011-0.0012)	0.045 (0.044-0.051)	0.82 (0.81-0.83)
Conspecific success, previous year	0.0076 (0.0071-0.0089)	0.24 (0.24-0.26)	0.015 (0.015-0.015)	0.012 (0.012-0.015)	0.67 (0.65-0.68)
Great tit abundance, current year	0.0090 (0.0088-0.012)	0.0067 (0.0061-0.0080)	0.0034 (0.0034-0.0036)	0.068 (0.068-0.075)	0.54 (0.53-0.55)
Great tit abundance, previous year	0.026 (0.014-0.040)	0.064 (0.051-0.073)	0.072 (0.071-0.074)	0.41 (0.41-0.41)	0.24 (0.21-0.26)

Table S3. Comparison of the variance component estimates in the quantitative genetic models (full models including the cross-sex additive genetic covariance) with varying prior specifications. Median estimates using the original prior and the range of median estimates (in parentheses; with the accuracy of two significant digits) using the nine alternative priors are presented for each variable.

**Response variable**	$V_{A♀}$	$V_{A♂}$	$Cov_{A♀♂}$	$V_{DOM♀}$	$V_{DOM♂}$	$V_{PI♀}$	$V_{PI♂}$	V_{PATCH}	V_{BOX}	V_R
Conspecific success, previous year	0.0073 (0.0071-0.0087)	0.035 (0.036-0.038)	$-3.0*10^{-5}$ ($-3.0*10^{-5}$-$1.1*10^{-5}$)	0.0064 (0.0059-0.0070)	0.095 (0.092-0.11)	0.0061 (0.0057-0.0067)	0.066 (0.066-0.073)	0.015 (0.015-0.015)	0.012 (0.012-0.015)	0.65 (0.63-0.66)
Great tit abundance, previous year	0.0035 (0.0032-0.0044)	0.018 (0.015-0.021)	$-4.6*10^{-5}$ ($-2.0*10^{-5}$-$5.0*10^{-6}$)	0.010 (0.0067-0.013)	0.018 (0.013-0.021)	0.012 (0.0079-0.018)	0.017 (0.013-0.020)	0.072 (0.071-0.073)	0.40 (0.40-0.40)	0.22 (0.20-0.25)

Section F. R syntax for fitting the models

R syntax for fitting the univariate GLMM estimating repeatability for each of the social cues (*ResponseVariable*):

*model1 <- MCMCglmm(ResponseVariable ~ StatusFemale * StatusMale * NestDate, random=~PermIndFemale + PermIndMale + Patch + Box, family="gaussian", prior=prior, data=data, nitt=2550000, burnin=50000, thin=500)*

R syntax for fitting the univariate GLMM estimating heritability, including the cross-sex additive genetic covariance, for each of the social cues (*ResponseVariable*):

*model2 <- MCMCglmm(ResponseVariable ~ StatusFemale * StatusMale * NestDate, random=~str(AddGenFemale + AddGenMale) + DomGenFemale + DomGenMale + PermIndFemale + PermIndMale + Patch + Box, ginverse=list(AddGenFemale=allA, AddGenMale=allA, DomGenFemale=Dinv_pruned, DomGenMale=Dinv_pruned), family="gaussian", prior=prior, data=data, nitt=2550000, burnin=50000, thin=500)*

Section G. Additional results

Repeatability analyses

Models with the full fixed effects structure

Table S4. Parameter estimates (posterior medians) and their 95% credibility intervals in the univariate GLMM estimating repeatability for the use of five social cues for breeding site choice in collared flycatcher: conspecific reproductive success in the previous year (n = 1395 breeding pairs), great tit abundance in the previous year (n = 1446), conspecific abundance in the current year (n = 1432), conspecific abundance in the previous year (n = 1430), and great tit abundance in the current year (n = 1430). The fixed effects include the "Status" (combination of dispersal status and age) for both females and males, the date of nest site choice "NestDate" and all their interactions (denoted with ":"). $V_{PI♀}$ and $V_{PI♂}$ are the female and male permanent individual variances, V_{PATCH} is the spatial variance across forest patches, V_{BOX} is the variance between nest boxes and V_R is the residual variance. Also the derived metrics total phenotypic variance V_P, total permanent individual variance $V_{PI\ total}$ and the repeatabilities for females $R_♀$ and males $R_♂$ and the total repeatability R_{total} are reported. N_{eff} is the effective MCMC sample size.

Response variable	Parameter	Median	95% CI	N_{eff}
Conspecific reproductive success in the previous year	*Fixed effects*			
	Intercept	0.11	-0.034 – 0.24	15000
	Status ♀, resident	0.0099	-0.17 – 0.19	15384
	Status ♀, young	0.081	-0.11 – 0.27	14878
	Status ♂, resident	0.12	-0.046 – 0.29	14893
	Status ♂, young	-0.035	-0.25 – 0.18	15643
	NestDate	-0.0058	-0.023 – 0.012	14932
	Status ♀, resident : Status ♂, resident	0.032	-0.23 – 0.29	15198
	Status ♀, young : Status ♂, resident	0.036	-0.26 – 0.33	14756
	Status ♀, resident : Status ♂, young	0.10	-0.30 – 0.48	14610
	Status ♀, young : Status ♂, young	-0.041	-0.38 – 0.29	15000
	Status ♀, resident : NestDate	-0.014	-0.047 – 0.018	14909
	Status ♀, young : NestDate	-0.0045	-0.032 – 0.024	15000
	Status ♂, resident : NestDate	0.021	-0.0079 – 0.050	14454
	Status ♂, young : NestDate	-0.0068	-0.045 – 0.031	15268
	Status ♀, resident : Status ♂, resident : NestDate	$1.0*10^{-4}$	-0.049 – 0.049	15000
	Status ♀, young : Status ♂, resident : NestDate	0.0019	-0.044 – 0.048	14934
	Status ♀, resident : Status ♂, young : NestDate	0.0022	-0.073 – 0.079	14437
	Status ♀, young : Status ♂, young : NestDate	0.0076	-0.045 – 0.060	15000
	Variance components			
	$V_{PI♀}$	0.0076	$1.8*10^{-5}$ – 0.063	15000
	$V_{PI♂}$	0.24	0.14 – 0.36	15479
	V_{PATCH}	0.015	0.0029 – 0.057	14672
	V_{BOX}	0.012	$3.2*10^{-5}$ – 0.080	14823
	V_R	0.67	0.58 – 0.78	15000
	Derived metrics			
	V_P	0.97	0.90 – 1.1	15000
	$V_{PI\ total}$	0.26	0.15 – 0.37	15433
	$R_♀$	0.0078	$1.9*10^{-5}$ – 0.064	15000
	$R_♂$	0.25	0.14 – 0.35	15500
	R_{total}	0.27	0.16 – 0.37	15470
Great tit abundance in the previous year	*Fixed effects*			
	Intercept	-0.14	-0.32 – 0.048	15003
	Status ♀, resident	-0.035	-0.17 – 0.095	15000
	Status ♀, young	0.047	-0.093 – 0.19	15000
	Status ♂, resident	-0.055	-0.18 – 0.068	15000
	Status ♂, young	-0.050	-0.20 – 0.11	15000

	NestDate	0.0076	-0.0050 – 0.020	14749
	Status ♀, resident : Status ♂, resident	0.012	-0.18 – 0.21	14786
	Status ♀, young : Status ♂, resident	-0.035	-0.26 – 0.19	15000
	Status ♀, resident : Status ♂, young	-0.14	-0.43 – 0.15	15000
	Status ♀, young : Status ♂, young	0.052	-0.19 – 0.30	14792
	Status ♀, resident : NestDate	0.0038	-0.020 – 0.028	14710
	Status ♀, young : NestDate	-0.0020	-0.023 – 0.019	14670
	Status ♂, resident : NestDate	0.059	-0.015 – 0.027	15556
	Status ♂, young : NestDate	$-8.5*10^{-5}$	-0.026 – 0.027	15312
	Status ♀, resident : Status ♂, resident : NestDate	-0.015	-0.051 – 0.022	14221
	Status ♀, young : Status ♂, resident : NestDate	$-2.7*10^{-4}$	-0.035 – 0.034	14776
	Status ♀, resident : Status ♂, young : NestDate	0.037	-0.020 – 0.095	15266
	Status ♀, young : Status ♂, young : NestDate	$5.7*10^{-4}$	-0.037 – 0.037	15233
	Variance components			
	$V_{\mathrm{PI}♀}$	0.026	$1.3*10^{-4}$ – 0.082	15000
	$V_{\mathrm{PI}♂}$	0.064	0.0093 – 0.12	15401
	V_{PATCH}	0.072	0.031 – 0.20	14789
	V_{BOX}	0.41	0.33 – 0.49	15000
	V_{R}	0.24	0.19 – 0.29	15000
	Derived metrics			
	V_{P}	0.82	0.74 – 0.96	14565
	$V_{\mathrm{PI\ total}}$	0.093	0.032 – 0.16	14774
	$R_{♀}$	0.032	$1.6*10^{-4}$ – 0.10	14608
	$R_{♂}$	0.078	0.011 – 0.15	15468
	R_{total}	0.11	0.038 – 0.20	14751
Conspecific abundance in the current year	*Fixed effects*			
	Intercept	-0.083	-0.25 – 0.091	15000
	Status ♀, resident	-0.10	-0.25 – 0.046	14923
	Status ♀, young	-0.20	-0.36 – -0.049	15274
	Status ♂, resident	-0.20	-0.35 – -0.060	14595
	Status ♂, young	-0.10	-0.28 – 0.072	15365
	NestDate	0.0043	-0.010 – 0.018	15000
	Status ♀, resident : Status ♂, resident	0.23	0.0070 – 0.45	15220
	Status ♀, young : Status ♂, resident	0.48	0.22 – 0.74	15000
	Status ♀, resident : Status ♂, young	0.32	-0.0073 – 0.64	14470
	Status ♀, young : Status ♂, young	0.26	-0.020 – 0.54	15000
	Status ♀, resident : NestDate	-0.018	-0.045 – 0.0090	14769
	Status ♀, young : NestDate	-0.047	-0.072 – -0.023	15295
	Status ♂, resident : NestDate	-0.029	-0.053 – -0.0043	14769
	Status ♂, young : NestDate	-0.023	-0.053 – 0.0079	14512
	Status ♀, resident : Status ♂, resident : NestDate	0.035	-0.0066 – 0.078	14768
	Status ♀, young : Status ♂, resident : NestDate	0.085	0.044 – 0.12	15514
	Status ♀, resident : Status ♂, young : NestDate	-0.026	-0.090 – 0.039	15120
	Status ♀, young : Status ♂, young : NestDate	0.011	-0.033 – 0.054	14854
	Variance components			
	$V_{\mathrm{PI}♀}$	0.016	$4.3*10^{-5}$ – 0.13	14781
	$V_{\mathrm{PI}♂}$	0.019	$4.2*10^{-5}$ – 0.14	14362
	V_{PATCH}	0.058	0.024 – 0.16	15278
	V_{BOX}	0.018	$4.5*10^{-5}$ – 0.11	15225
	V_{R}	0.59	0.45 – 0.68	15370
	Derived metrics			
	V_{P}	0.74	0.67 – 0.85	15277
	$V_{\mathrm{PI\ total}}$	0.050	0.0021 – 0.19	15687
	$R_{♀}$	0.021	$5.8*10^{-5}$ – 0.17	14800
	$R_{♂}$	0.025	$5.7*10^{-5}$ – 0.19	14355
	R_{total}	0.067	0.0028 – 0.26	15694
Conspecific abundance in the previous year	*Fixed effects*			
	Intercept	0.19	0.079 – 0.30	14799
	Status ♀, resident	0.20	0.021 – 0.37	14730
	Status ♀, young	-0.11	-0.29 – 0.076	14277

	Status ♂, resident	0.17	-6.4*10^{-4} – 0.33	14313
	Status ♂, young	-0.077	-0.28 – 0.13	15000
	NestDate	-0.0024	-0.019 – 0.014	15000
	Status ♀, resident : Status ♂, resident	-0.097	-0.36 – 0.16	14517
	Status ♀, young : Status ♂, resident	0.11	-0.19 – 0.41	14708
	Status ♀, resident : Status ♂, young	0.0052	-0.38 – 0.40	14149
	Status ♀, young : Status ♂, young	0.026	-0.30 – 0.35	14746
	Status ♀, resident : NestDate	0.017	-0.015 – 0.049	15000
	Status ♀, young : NestDate	0.0095	-0.018 – 0.037	15000
	Status ♂, resident : NestDate	-0.014	-0.042 – 0.014	15545
	Status ♂, young : NestDate	0.0052	-0.031 – 0.041	14349
	Status ♀, resident : Status ♂, resident : NestDate	-0.037	-0.085 – 0.013	15386
	Status ♀, young : Status ♂, resident : NestDate	0.0021	-0.044 – 0.047	14714
	Status ♀, resident : Status ♂, young : NestDate	-0.045	-0.12 – 0.029	15000
	Status ♀, young : Status ♂, young : NestDate	-0.017	-0.067 – 0.032	15000
	Variance components			
	$V_{PI♀}$	0.016	4.8*10^{-5} – 0.090	15972
	$V_{PI♂}$	0.015	3.9*10^{-5} – 0.080	14624
	V_{PATCH}	0.0012	2.5*10^{-6} – 0.014	14492
	V_{BOX}	0.045	6.8*10^{-4} – 0.11	14797
	V_R	0.82	0.72 – 0.92	15000
	Derived metrics			
	V_P	0.92	0.86 – 0.99	15000
	$V_{PI\ total}$	0.039	0.0022 – 0.13	15000
	$R_{♀}$	0.018	5.3*10^{-5} – 0.097	15952
	$R_{♂}$	0.016	4.3*10^{-5} – 0.086	14621
	R_{total}	0.043	0.0023 – 0.13	15000
Great tit abundance in the current year	*Fixed effects*			
	Intercept	0.19	0.092 – 0.28	15000
	Status ♀, resident	-0.044	-0.19 – 0.099	15240
	Status ♀, young	-0.026	-0.18 – 0.13	15000
	Status ♂, resident	-0.052	-0.19 – 0.087	16969
	Status ♂, young	0.10	-0.069 – 0.28	15000
	NestDate	0.0020	-0.012 – 0.016	15096
	Status ♀, resident : Status ♂, resident	-0.0058	-0.22 – 0.22	15000
	Status ♀, young : Status ♂, resident	0.032	-0.22 – 0.28	14739
	Status ♀, resident : Status ♂, young	-0.14	-0.46 – 0.18	15000
	Status ♀, young : Status ♂, young	-0.077	-0.35 – 0.19	15325
	Status ♀, resident : NestDate	0.0045	-0.022 – 0.031	15063
	Status ♀, young : NestDate	-0.019	-0.042 – 0.0053	14630
	Status ♂, resident : NestDate	0.0024	-0.021 – 0.026	15221
	Status ♂, young : NestDate	-0.046	-0.076 – -0.016	14483
	Status ♀, resident : Status ♂, resident : NestDate	-0.017	-0.058 – 0.023	14968
	Status ♀, young : Status ♂, resident : NestDate	-0.0019	-0.040 – 0.037	15000
	Status ♀, resident : Status ♂, young : NestDate	0.059	-0.0020 – 0.12	15000
	Status ♀, young : Status ♂, young : NestDate	0.061	0.019 – 0.10	14673
	Variance components			
	$V_{PI♀}$	0.0090	2.4*10^{-5} – 0.061	15000
	$V_{PI♂}$	0.0067	1.4*10^{-5} – 0.046	15215
	V_{PATCH}	0.0034	1.1*10^{-5} – 0.027	15000
	V_{BOX}	0.068	0.012 – 0.13	15000
	V_R	0.54	0.47 – 0.62	15000
	Derived metrics			
	V_P	0.64	0.60 – 0.70	15000
	$V_{PI\ total}$	0.022	9.9*10^{-4} – 0.080	15221
	$R_{♀}$	0.014	3.6*10^{-5} – 0.093	15000
	$R_{♂}$	0.010	2.2*10^{-5} – 0.070	15210
	R_{total}	0.033	0.0015 – 0.12	15003

Models with only the intercept as the fixed effect

Table S5. Parameter estimates (posterior medians) and their 95% credibility intervals in the univariate GLMM estimating repeatability for the use of five social cues for breeding site choice in collared flycatcher: conspecific reproductive success in the previous year (n = 1395 breeding pairs), great tit abundance in the previous year (n = 1446), conspecific abundance in the current year (n = 1432), conspecific abundance in the previous year (n = 1430), and great tit abundance in the current year (n = 1430). Only the intercept is included as a fixed effect. $V_{PI♀}$ and $V_{PI♂}$ are the female and male permanent individual variances, V_{PATCH} is the spatial variance across forest patches, V_{BOX} is the variance between nest boxes and V_R is the residual variance. Also the derived metrics total phenotypic variance V_P, total permanent individual variance $V_{PI\ total}$ and the repeatabilities for females $R_♀$ and males $R_♂$ and the total repeatability R_{total} are reported. N_{eff} is the effective MCMC sample size.

**Response variable**	**Parameter**	**Median**	**95% CI**	**N_{eff}**
Conspecific reproductive success in the previous year	*Fixed effects*			
	Intercept	0.16	0.054 – 0.25	15000
	Variance components			
	$V_{PI♀}$	0.0073	$1.8*10^{-5}$ – 0.061	15000
	$V_{PI♂}$	0.26	0.16 – 0.37	14351
	V_{PATCH}	0.016	0.0034 – 0.057	14690
	V_{BOX}	0.011	$3.1*10^{-5}$ – 0.074	15000
	V_R	0.66	0.57 – 0.76	14613
	Derived metrics			
	V_P	0.98	0.90 – 1.1	14755
	$V_{PI\ total}$	0.28	0.17 – 0.38	14729
	$R_♀$	0.0074	$1.9*10^{-5}$ – 0.061	15210
	$R_♂$	0.27	0.17 – 0.37	14641
	R_{total}	0.28	0.18 – 0.38	14656
Great tit abundance in the previous year	*Fixed effects*			
	Intercept	-0.16	-0.32 – 0.015	15000
	Variance components			
	$V_{PI♀}$	0.023	$1.5*10^{-4}$ – 0.075	14746
	$V_{PI♂}$	0.065	0.011 – 0.12	15000
	V_{PATCH}	0.073	0.031 – 0.20	14700
	V_{BOX}	0.42	0.34 – 0.50	15000
	V_R	0.23	0.18 – 0.29	16055
	Derived metrics			
	V_P	0.82	0.74 – 0.97	14541
	$V_{PI\ total}$	0.091	0.032 – 0.16	15000
	$R_♀$	0.028	$1.8*10^{-4}$ – 0.091	14703
	$R_♂$	0.079	0.013 – 0.15	15000
	R_{total}	0.11	0.038 – 0.19	15240
Conspecific abundance in the current year	*Fixed effects*			
	Intercept	-0.18	-0.33 – -0.021	15076
	Variance components			
	$V_{PI♀}$	0.020	$4.8*10^{-5}$ – 0.15	15000
	$V_{PI♂}$	0.060	$1.8*10^{-4}$ – 0.23	14520
	V_{PATCH}	0.061	0.025 – 0.17	15000
	V_{BOX}	0.013	$2.5*10^{-5}$ – 0.097	15000
	V_R	0.58	0.42 – 0.70	14377
	Derived metrics			
	V_P	0.77	0.70 – 0.90	15000
	$V_{PI\ total}$	0.10	0.0050 – 0.29	14304
	$R_♀$	0.026	$6.3*10^{-5}$ – 0.20	15000
	$R_♂$	0.077	$2.4*10^{-4}$ – 0.29	14555
	R_{total}	0.13	0.0066 – 0.36	14366
Conspecific abundance in the	*Fixed effects*			

previous year	Intercept	0.26	0.19 – 0.32	15668
	Variance components			
	$V_{PI♀}$	0.017	$4.6*10^{-5}$ – 0.091	15241
	$V_{PI♂}$	0.013	$2.7*10^{-5}$ – 0.079	14776
	V_{PATCH}	$9.2*10^{-4}$	$1.7*10^{-6}$ – 0.013	15000
	V_{BOX}	0.049	0.0012 – 0.12	15000
	V_{R}	0.84	0.73 – 0.94	15000
	Derived metrics			
	V_{P}	0.94	0.87 – 1.0	14705
	$V_{PI\ total}$	0.039	0.0021 – 0.13	15307
	$R_{♀}$	0.018	$4.9*10^{-5}$ – 0.097	15232
	$R_{♂}$	0.014	$2.9*10^{-5}$ – 0.085	14769
	R_{total}	0.042	0.0022 – 0.13	15281
Great tit abundance in the current year	*Fixed effects*			
	Intercept	0.16	0.095 – 0.22	15000
	Variance components			
	$V_{PI♀}$	0.0094	$2.4*10^{-5}$ – 0.063	13735
	$V_{PI♂}$	0.0071	$1.8*10^{-5}$ – 0.049	15273
	V_{PATCH}	0.0025	$6.4*10^{-6}$ – 0.023	14980
	V_{BOX}	0.064	0.0092 – 0.12	14665
	V_{R}	0.55	0.47 – 0.62	15000
	Derived metrics			
	V_{P}	0.65	0.60 – 0.70	15000
	$V_{PI\ total}$	0.022	$9.8*10^{-4}$ – 0.083	15000
	$R_{♀}$	0.015	$3.7*10^{-5}$ – 0.097	14086
	$R_{♂}$	0.011	$2.8*10^{-5}$ – 0.076	15266
	R_{total}	0.035	0.0015 – 0.13	15000

Quantitative genetic analyses

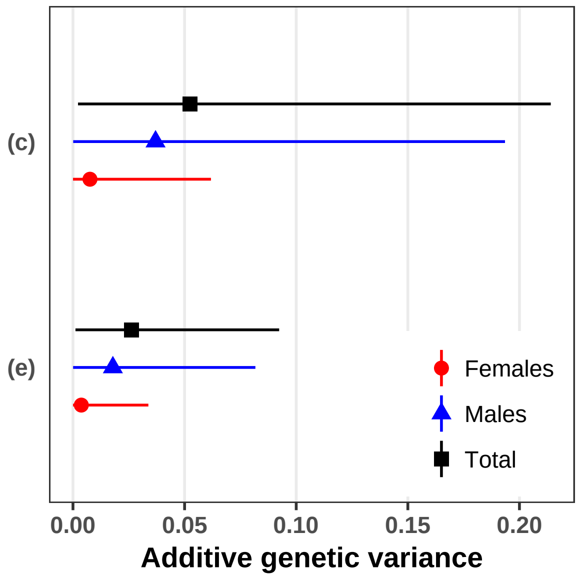

Figure S3. Estimates of female (red circles), male (blue triangles) and total (black squares) additive genetic variances (median ± 95% CI) in the use of (c) conspecific success in the previous year and (e) great tit abundance in the previous year as social cues for breeding site choice by collared flycatchers, based on the models with the full fixed effects structure, but excluding the cross-sex additive genetic covariance (see Table S7).

Models with the full fixed and random effects structure

Table S6. Parameter estimates (posterior medians) and their 95% credibility intervals in the univariate GLMM estimating additive genetic variance and heritability for the use of two social cues for breeding site choice in collared flycatcher: conspecific reproductive success in the previous year (n = 1395 breeding pairs), great tit abundance in the previous year (n = 1446). The fixed effects include the "Status" (combination of dispersal status and age) for both females and males, the date of nest site choice "NestDate" and all their interactions (denoted with ":"). $V_{A♀}$ and $V_{A♂}$ are the female and male additive genetic variances, $Cov_{A♀♂}$ is the female-male additive genetic covariance, $V_{DOM♀}$ and $V_{DOM♂}$ are the female and male dominance genetic variances, $V_{PI♀}$ and $V_{PI♂}$ are the female and male permanent individual variances, V_{PATCH} is the spatial variance across forest patches, V_{BOX} is the variance between nest boxes and V_R is the residual variance. Also the derived metrics total additive genetic variance $V_{A\,total}$, total phenotypic variance V_P and female $h^2_♀$, male $h^2_♂$ and total heritabilities T^2 are reported. N_{eff} is the effective MCMC sample size.

Response variable	Parameter	Median	95% CI	N_{eff}
Conspecific reproductive success in the previous year	*Fixed effects*			
	Intercept	0.10	-0.036 – 0.24	14953
	Status ♀, resident	0.0060	-0.17 – 0.18	14553
	Status ♀, young	0.077	-0.11 – 0.27	16072
	Status ♂, resident	0.12	-0.051 – 0.29	14847
	Status ♂, young	-0.028	-0.24 – 0.19	15000
	NestDate	-0.0063	-0.024 – 0.011	15136
	Status ♀, resident : Status ♂, resident	0.041	-0.21 – 0.30	15948
	Status ♀, young : Status ♂, resident	0.049	-0.26 – 0.35	15000
	Status ♀, resident : Status ♂, young	0.10	-0.28 – 0.49	15000
	Status ♀, young : Status ♂, young	-0.045	-0.38 – 0.29	15000
	Status ♀, resident : NestDate	-0.013	-0.047 – 0.019	14758
	Status ♀, young : NestDate	-0.0047	-0.034 – 0.024	15000
	Status ♂, resident : NestDate	0.021	-0.0077 – 0.049	15000
	Status ♂, young : NestDate	-0.0066	-0.045 – 0.032	15000
	Status ♀, resident : Status ♂, resident : NestDate	$6.1*10^{-4}$	-0.048 – 0.049	15112
	Status ♀, young : Status ♂, resident : NestDate	0.0034	-0.043 – 0.050	14555
	Status ♀, resident : Status ♂, young : NestDate	0.0029	-0.071 – 0.081	15094
	Status ♀, young : Status ♂, young : NestDate	0.0078	-0.044 – 0.059	15000
	Variance components			
	$V_{A♀}$	0.0073	$1.6*10^{-5}$ – 0.060	15228
	$V_{A♂}$	0.035	$8.0*10^{-5}$ – 0.19	14754
	$Cov_{A♀♂}$	$-3.0*10^{-5}$	-0.031 – 0.026	14354
	$V_{DOM♀}$	0.0064	$1.6*10^{-5}$ – 0.056	15000
	$V_{DOM♂}$	0.095	$3.6*10^{-4}$ – 0.28	14734
	$V_{PI♀}$	0.0061	$1.2*10^{-5}$ – 0.054	15723
	$V_{PI♂}$	0.066	$1.9*10^{-4}$ – 0.27	15000
	V_{PATCH}	0.015	0.0028 – 0.057	14749
	V_{BOX}	0.012	$3.0*10^{-5}$ – 0.079	14309
	V_R	0.65	0.56 – 0.76	15703
	Derived metrics			
	V_P	0.98	0.90 – 1.1	15944
	$V_{A\,total}$	0.048	0.0016 – 0.22	14764
	$h^2_♀$	0.0074	$1.6*10^{-5}$ – 0.061	15222
	$h^2_♂$	0.036	$8.3*10^{-5}$ – 0.19	14747
	T^2	0.049	0.0016 – 0.22	14762
Great tit abundance in the previous year	*Fixed effects*			
	Intercept	-0.13	-0.31 – 0.057	15261
	Status ♀, resident	-0.038	-0.17 – 0.094	14632
	Status ♀, young	0.050	-0.090 – 0.19	15000
	Status ♂, resident	-0.057	-0.18 – 0.065	15140

Status ♂, young	-0.048	-0.20 – 0.11	14029
NestDate	0.0075	-0.0051 – 0.020	15000
Status ♀, resident : Status ♂, resident	0.017	-0.18 – 0.21	14918
Status ♀, young : Status ♂, resident	-0.042	-0.27 – 0.19	14943
Status ♀, resident : Status ♂, young	-0.13	-0.42 – 0.16	14794
Status ♀, young : Status ♂, young	0.050	-0.20 – 0.30	14909
Status ♀, resident : NestDate	0.0042	-0.020 – 0.028	14780
Status ♀, young : NestDate	-0.0018	-0.023 – 0.019	15073
Status ♂, resident : NestDate	0.0059	-0.015 – 0.027	15000
Status ♂, young : NestDate	$-6.7*10^{-5}$	-0.027 – 0.027	14938
Status ♀, resident : Status ♂, resident : NestDate	-0.016	-0.052 – 0.020	14517
Status ♀, young : Status ♂, resident : NestDate	$-9.2*10^{-4}$	-0.036 – 0.033	15000
Status ♀, resident : Status ♂, young : NestDate	0.037	-0.021 – 0.094	15154
Status ♀, young : Status ♂, young : NestDate	$-1.6*10^{-4}$	-0.038 – 0.037	15803
Variance components			
$V_{A♀}$	0.0035	$7.3*10^{-6}$ – 0.033	15302
$V_{A♂}$	0.018	$5.2*10^{-5}$ – 0.081	14340
$Cov_{A♀♂}$	$-4.6*10^{-5}$	-0.016 – 0.012	15000
$V_{DOM♀}$	0.010	$2.7*10^{-5}$ – 0.060	15000
$V_{DOM♂}$	0.018	$3.7*10^{-5}$ – 0.088	15000
$V_{PI♀}$	0.012	$3.7*10^{-5}$ – 0.065	14919
$V_{PI♂}$	0.017	$4.2*10^{-5}$ – 0.087	15000
V_{PATCH}	0.072	0.031 – 0.21	14874
V_{BOX}	0.40	0.32 – 0.48	14623
V_R	0.22	0.18 – 0.28	15000
Derived metrics			
V_P	0.82	0.74 – 0.96	15991
$V_{A\ total}$	0.024	$8.6*10^{-4}$ – 0.095	15000
$h^2_♀$	0.0043	$9.1*10^{-6}$ – 0.041	15296
$h^2_♂$	0.022	$6.3*10^{-5}$ – 0.10	14341
T^2	0.029	0.0011 – 0.12	15000

Models with the full fixed effects structure, but excluding the cross-sex additive genetic covariance

Table S7. Parameter estimates (posterior medians) and their 95% credibility intervals in the univariate GLMM estimating additive genetic variance and heritability for the use of two social cues for breeding site choice in collared flycatcher: conspecific reproductive success in the previous year (n = 1395 breeding pairs), great tit abundance in the previous year (n = 1446). Compared to the model in Table S6, this model excludes the additive genetic covariance between females and males. The fixed effects include the “Status” (combination of dispersal status and age) for both females and males, the date of nest site choice “NestDate” and all their interactions (denoted with “:”). $V_{A♀}$ and $V_{A♂}$ are the female and male additive genetic variances, $V_{DOM♀}$ and $V_{DOM♂}$ are the female and male dominance genetic variances, $V_{PI♀}$ and $V_{PI♂}$ are the female and male permanent individual variances, V_{PATCH} is the spatial variance across forest patches, V_{BOX} is the variance between nest boxes and V_R is the residual variance. Also the derived metrics total additive genetic variance $V_{A\ total}$, total phenotypic variance V_P and female $h^2_♀$, male $h^2_♂$ and total heritabilities T^2 are reported. N_{eff} is the effective MCMC sample size.

**Response variable**	**Parameter**	**Median**	**95% CI**	N_{eff}
Conspecific reproductive success in the previous year	*Fixed effects*			
	Intercept	0.10	-0.035 – 0.24	16367
	Status ♀, resident	0.0059	-0.17 – 0.18	15230
	Status ♀, young	0.078	-0.11 – 0.27	15000
	Status ♂, resident	0.12	-0.050 – 0.29	15541
	Status ♂, young	-0.030	-0.24 – 0.18	15000
	NestDate	-0.0062	-0.024 – 0.011	14640
	Status ♀, resident : Status ♂, resident	0.040	-0.22 – 0.30	14857
	Status ♀, young : Status ♂, resident	0.050	-0.26 – 0.36	15119
	Status ♀, resident : Status ♂, young	0.098	-0.29 – 0.49	15627
	Status ♀, young : Status ♂, young	-0.048	-0.38 – 0.29	15000
	Status ♀, resident : NestDate	-0.014	-0.046 – 0.019	15000
	Status ♀, young : NestDate	-0.0048	-0.033 – 0.023	15000
	Status ♂, resident : NestDate	0.021	-0.0074 – 0.049	14728
	Status ♂, young : NestDate	-0.0061	-0.044 – 0.032	14867
	Status ♀, resident : Status ♂, resident : NestDate	$6.0*10^{-4}$	-0.048 – 0.049	15000
	Status ♀, young : Status ♂, resident : NestDate	0.0034	-0.044 – 0.049	14773
	Status ♀, resident : Status ♂, young : NestDate	0.0033	-0.074 – 0.080	15249
	Status ♀, young : Status ♂, young : NestDate	0.0081	-0.044 – 0.059	14693
	Variance components			
	$V_{A♀}$	0.0076	$1.6*10^{-5}$ – 0.062	15444
	$V_{A♂}$	0.037	$9.9*10^{-5}$ – 0.19	16382
	$V_{DOM♀}$	0.0064	$1.3*10^{-5}$ – 0.055	15000
	$V_{DOM♂}$	0.093	$3.5*10^{-4}$ – 0.28	15011
	$V_{PI♀}$	0.0061	$1.4*10^{-5}$ – 0.053	15000
	$V_{PI♂}$	0.067	$1.6*10^{-4}$ – 0.27	15000
	V_{PATCH}	0.015	0.0028 – 0.058	14182
	V_{BOX}	0.012	$2.9*10^{-5}$ – 0.078	15000
	V_R	0.66	0.56 – 0.76	13902
	Derived metrics			
	V_P	0.98	0.90 – 1.1	15247
	$V_{A\ total}$	0.052	0.0022 – 0.21	15000
	$h^2_♀$	0.0077	$1.6*10^{-5}$ – 0.063	15430
	$h^2_♂$	0.038	$9.9*10^{-5}$ – 0.20	16501
	T^2	0.054	0.0023 – 0.22	16564
Great tit abundance in the previous year	*Fixed effects*			
	Intercept	-0.13	-0.31 – 0.055	15114
	Status ♀, resident	-0.039	-0.17 – 0.092	14785
	Status ♀, young	0.049	-0.092 – 0.19	15000
	Status ♂, resident	-0.057	-0.18 – 0.064	14924

Status ♂, young	-0.050	-0.21 – 0.11	14922
NestDate	0.0075	-0.0051 – 0.020	15633
Status ♀, resident : Status ♂, resident	0.019	-0.17 – 0.21	15000
Status ♀, young : Status ♂, resident	-0.040	-0.27 – 0.19	15509
Status ♀, resident : Status ♂, young	-0.13	-0.42 – 0.15	15000
Status ♀, young : Status ♂, young	0.053	-0.20 – 0.30	14785
Status ♀, resident : NestDate	0.0042	-0.020 – 0.028	14734
Status ♀, young : NestDate	-0.0018	-0.023 – 0.019	15000
Status ♂, resident : NestDate	0.0061	-0.015 – 0.027	14653
Status ♂, young : NestDate	$-2.2*10^{-4}$	-0.027 – 0.027	15000
Status ♀, resident : Status ♂, resident : NestDate	-0.016	-0.053 – 0.019	15000
Status ♀, young : Status ♂, resident : NestDate	-0.0010	-0.035 – 0.034	15000
Status ♀, resident : Status ♂, young : NestDate	0.037	-0.020 – 0.094	14751
Status ♀, young : Status ♂, young : NestDate	$3.1*10^{-5}$	-0.037 – 0.037	15000
Variance components			
$V_{A♀}$	0.0037	$7.8*10^{-6}$ – 0.034	15549
$V_{A♂}$	0.018	$5.7*10^{-5}$ – 0.082	15038
$V_{DOM♀}$	0.010	$2.6*10^{-5}$ – 0.060	15296
$V_{DOM♂}$	0.018	$4.5*10^{-5}$ – 0.089	14998
$V_{PI♀}$	0.012	$2.9*10^{-5}$ – 0.066	15000
$V_{PI♂}$	0.018	$4.1*10^{-5}$ – 0.089	14790
V_{PATCH}	0.072	0.031 – 0.21	15391
V_{BOX}	0.40	0.32 – 0.48	15153
V_R	0.22	0.18 – 0.27	15207
Derived metrics			
V_P	0.82	0.74 – 0.96	14659
$V_{A\ total}$	0.026	0.0011 – 0.092	15062
$h^2_♀$	0.0045	$9.4*10^{-6}$ – 0.041	15583
$h^2_♂$	0.022	$7.1*10^{-5}$ – 0.10	15142
T^2	0.032	0.0013 – 0.11	15054

Models with the full fixed effects structure, but excluding the cross-sex additive genetic covariance and the dominance genetic random effects

Table S8. Parameter estimates (posterior medians) and their 95% credibility intervals in the univariate GLMM estimating additive genetic variance and heritability for the use of two social cues for breeding site choice in collared flycatcher: conspecific reproductive success in the previous year (n = 1395 breeding pairs), great tit abundance in the previous year (n = 1446). Compared to the model in Table S6, this model excludes the additive genetic covariance between females and males and the dominance genetic effects for both sexes. The fixed effects include the "Status" (combination of dispersal status and age) for both females and males, the date of nest site choice "NestDate" and all their interactions (denoted with ":"). $V_{A♀}$ and $V_{A♂}$ are the female and male additive genetic variances, $V_{PI♀}$ and $V_{PI♂}$ are the female and male permanent individual variances, V_{PATCH} is the spatial variance across forest patches, V_{BOX} is the variance between nest boxes and V_R is the residual variance. Also the derived metrics total additive genetic variance $V_{A\,total}$, total phenotypic variance V_P and female $h^2_{♀}$, male $h^2_{♂}$ and total heritabilities T^2 are reported. N_{eff} is the effective MCMC sample size.

Response variable	Parameter	Median	95% CI	N_{eff}
Conspecific reproductive success in the previous year	*Fixed effects*			
	Intercept	0.10	-0.039 – 0.24	15000
	Status ♀, resident	0.0090	-0.17 – 0.19	15000
	Status ♀, young	0.078	-0.12 – 0.27	15505
	Status ♂, resident	0.12	-0.054 – 0.29	14705
	Status ♂, young	-0.028	-0.24 – 0.18	14707
	NestDate	-0.0062	-0.024 – 0.011	14677
	Status ♀, resident : Status ♂, resident	0.038	-0.22 – 0.30	15000
	Status ♀, young : Status ♂, resident	0.050	-0.26 – 0.36	15292
	Status ♀, resident : Status ♂, young	0.10	-0.28 – 0.49	15000
	Status ♀, young : Status ♂, young	-0.048	-0.38 – 0.29	15000
	Status ♀, resident : NestDate	-0.014	-0.046 – 0.019	14630
	Status ♀, young : NestDate	-0.0048	-0.032 – 0.024	15000
	Status ♂, resident : NestDate	0.021	-0.0081 – 0.049	15000
	Status ♂, young : NestDate	-0.0066	-0.044 – 0.031	15155
	Status ♀, resident : Status ♂, resident : NestDate	$2.0*10^{-4}$	-0.049 – 0.050	14790
	Status ♀, young : Status ♂, resident : NestDate	0.0033	-0.043 – 0.050	15000
	Status ♀, resident : Status ♂, young : NestDate	0.0033	-0.074 – 0.079	14714
	Status ♀, young : Status ♂, young : NestDate	0.0082	-0.043 – 0.059	15000
	Variance components			
	$V_{A♀}$	0.0091	$1.8*10^{-5}$ – 0.068	15000
	$V_{A♂}$	0.057	$1.6*10^{-4}$ – 0.24	14785
	$V_{PI♀}$	0.0068	$1.6*10^{-5}$ – 0.060	15000
	$V_{PI♂}$	0.17	0.0039 – 0.32	14116
	V_{PATCH}	0.015	0.0028 – 0.057	14434
	V_{BOX}	0.013	$3.4*10^{-5}$ – 0.081	15235
	V_R	0.67	0.57 – 0.77	15000
	Derived metrics			
	V_P	0.98	0.90 – 1.1	15000
	$V_{A\,total}$	0.074	0.0031 – 0.26	15000
	$h^2_{♀}$	0.0093	$1.9*10^{-5}$ – 0.069	15000
	$h^2_{♂}$	0.058	$1.6*10^{-4}$ – 0.24	14783
	T^2	0.076	0.0032 – 0.26	15000
Great tit abundance in the previous year	*Fixed effects*			
	Intercept	-0.13	-0.31 – 0.057	14795
	Status ♀, resident	-0.036	-0.17 – 0.093	15000
	Status ♀, young	0.049	-0.093 – 0.19	15000
	Status ♂, resident	-0.057	-0.18 – 0.068	14718

Status ♂, young	-0.050	-0.20 – 0.11	15439
NestDate	0.0076	-0.0048 – 0.020	15000
Status ♀, resident : Status ♂, resident	0.015	-0.18 – 0.21	15513
Status ♀, young : Status ♂, resident	-0.038	-0.27 – 0.19	15000
Status ♀, resident : Status ♂, young	-0.14	-0.42 – 0.14	15000
Status ♀, young : Status ♂, young	0.052	-0.19 – 0.30	15000
Status ♀, resident : NestDate	0.0043	-0.020 – 0.028	14764
Status ♀, young : NestDate	-0.0019	-0.023 – 0.019	14731
Status ♂, resident : NestDate	0.0058	-0.015 – 0.027	15582
Status ♂, young : NestDate	-4.9×10^{-4}	-0.027 – 0.026	14149
Status ♀, resident : Status ♂, resident : NestDate	-0.016	-0.052 – 0.020	14797
Status ♀, young : Status ♂, resident : NestDate	-9.5×10^{-4}	-0.035 – 0.034	14727
Status ♀, resident : Status ♂, young : NestDate	0.038	-0.021 – 0.095	15000
Status ♀, young : Status ♂, young : NestDate	4.1×10^{-5}	-0.037 – 0.037	14629
Variance components			
$V_{A♀}$	0.0043	1.1×10^{-5} – 0.038	15000
$V_{A♂}$	0.027	1.2×10^{-4} – 0.093	15000
$V_{PI♀}$	0.021	8.7×10^{-5} – 0.076	14585
$V_{PI♂}$	0.034	1.2×10^{-4} – 0.10	15000
V_{PATCH}	0.074	0.031 – 0.20	13764
V_{BOX}	0.40	0.33 – 0.49	15000
V_R	0.23	0.18 – 0.28	15313
Derived metrics			
V_P	0.82	0.74 – 0.95	13779
$V_{A\ total}$	0.036	0.0018 – 0.11	15418
$h^2_♀$	0.0053	1.3×10^{-5} – 0.046	15000
$h^2_♂$	0.033	1.4×10^{-4} – 0.11	15000
T^2	0.044	0.0021 – 0.13	15411

Models with only the intercept as the fixed effect and excluding the cross-sex additive genetic covariance

Table S9. Parameter estimates (posterior medians) and their 95% credibility intervals in the univariate GLMM estimating additive genetic variance and heritability for the use of two social cues for breeding site choice in collared flycatcher: conspecific reproductive success in the previous year (n = 1395 breeding pairs), great tit abundance in the previous year (n = 1446). Compared to the model in Table S7, this model includes only the intercept as a fixed effect. $V_{A♀}$ and $V_{A♂}$ are the female and male additive genetic variances, $V_{DOM♀}$ and $V_{DOM♂}$ are the female and male dominance genetic variances, $V_{PI♀}$ and $V_{PI♂}$ are the female and male permanent individual variances, V_{PATCH} is the spatial variance across forest patches, V_{BOX} is the variance between nest boxes and V_R is the residual variance. Also the derived metrics total additive genetic variance $V_{A\ total}$, total phenotypic variance V_P and female $h^2_♀$, male $h^2_♂$and total heritabilities T^2 are reported. N_{eff} is the effective MCMC sample size.

Response variable	Parameter	Median	95% CI	N_{eff}
Conspecific reproductive success in the previous year	*Fixed effects*			
	Intercept	0.15	0.051 – 0.25	15236
	Variance components			
	$V_{A♀}$	0.0079	$2.0*10^{-5}$ – 0.064	15000
	$V_{A♂}$	0.037	$9.0*10^{-5}$ – 0.20	14445
	$V_{DOM♀}$	0.0057	$1.4*10^{-5}$ – 0.054	14995
	$V_{DOM♂}$	0.10	$3.7*10^{-4}$ – 0.29	16168
	$V_{PI♀}$	0.0056	$1.4*10^{-5}$ – 0.054	14578
	$V_{PI♂}$	0.076	$1.9*10^{-4}$ – 0.28	15000
	V_{PATCH}	0.016	0.0030 – 0.058	15000
	V_{BOX}	0.011	$2.6*10^{-5}$ – 0.074	14594
	V_R	0.64	0.55 – 0.74	15222
	Derived metrics			
	V_P	0.98	0.91 – 1.1	14628
	$V_{A\ total}$	0.053	0.0020 – 0.22	14635
	$h^2_♀$	0.0081	$2.0*10^{-5}$ – 0.065	14851
	$h^2_♂$	0.037	$8.8*10^{-5}$ – 0.20	14479
	T^2	0.054	0.0021 – 0.22	14646
Great tit abundance in the previous year	*Fixed effects*			
	Intercept	-0.15	-0.32 – 0.022	15298
	Variance components			
	$V_{A♀}$	0.0037	$8.2*10^{-6}$ – 0.033	15560
	$V_{A♂}$	0.017	$5.2*10^{-5}$ – 0.080	15000
	$V_{DOM♀}$	0.0090	$2.3*10^{-5}$ – 0.056	15000
	$V_{DOM♂}$	0.018	$4.3*10^{-5}$ – 0.089	15000
	$V_{PI♀}$	0.011	$2.7*10^{-5}$ – 0.061	14724
	$V_{PI♂}$	0.018	$5.4*10^{-5}$ – 0.087	15000
	V_{PATCH}	0.073	0.032 – 0.20	13984
	V_{BOX}	0.41	0.33 – 0.49	15228
	V_R	0.22	0.17 – 0.27	15270
	Derived metrics			
	V_P	0.82	0.74 – 0.96	14246
	$V_{A\ total}$	0.026	$9.9*10^{-4}$ – 0.090	15000
	$h^2_♀$	0.0045	$9.8*10^{-6}$ – 0.040	15542
	$h^2_♂$	0.021	$6.2*10^{-5}$ – 0.097	15000
	T^2	0.031	0.0012 – 0.11	15000

Comparison of repeatability estimates between the repeatability and the quantitative genetic models

To illustrate that the quantitative genetic models (with full fixed effects structure and including the dominance genetic variances but excluding the cross-sex additive genetic covariance, see Table S7) give similar repeatability estimates as the more simple repeatability models (full fixed effects structure, see Table S4), we compared the repeatability estimates derived from these models in Table S10. Similar repeatability estimates suggest that the additive genetic, dominance genetic and (other) permanent individual variance component estimates in the quantitative genetic models are not biased low, and thus show that the data is adequate for fitting the quantitative genetic models. For the quantitative genetic models, the permanent individual variance was derived as the sum of additive genetic, dominance genetic and (other) permanent individual variance components.

Table S10. Comparison of the repeatability estimates derived from the repeatability and the quantitative genetic models. $R_{♀}$ is the repeatability for females, $R_{♂}$ the repeatability for males and R_{total} the total repeatability. Median estimates with 95% CIs in parentheses are reported.

Response variable	Parameter	Repeatability model	Genetic model
Conspecific reproductive success in the previous year	$R_{♀}$	0.0078 ($1.9*10^{-5}$ – 0.064)	0.034 (0.0037 – 0.099)
	$R_{♂}$	0.25 (0.14 – 0.35)	0.25 (0.15 – 0.35)
	R_{total}	0.27 (0.16 – 0.37)	0.29 (0.18 – 0.39)
Great tit abundance in the previous year	$R_{♀}$	0.032 ($1.6*10^{-4}$ – 0.10)	0.048 (0.0074 – 0.11)
	$R_{♂}$	0.078 (0.011 – 0.15)	0.091 (0.032 – 0.16)
	R_{total}	0.11 (0.038 – 0.20)	0.14 (0.069 – 0.23)

Section H. References

Kivelä, S. M., J.-T. Seppänen, O. Ovaskainen, B. Doligez, L. Gustafsson, M. Mönkkönen, and J. T. Forsman. 2014. The past and the present in decision-making: the use of conspecific and heterospecific cues in nest site selection. Ecology 95:3428–3439.

Wilson, A. J., D. Réale, M. N. Clements, M. M. Morrissey, E. Postma, C. A. Walling, L. E. B. Kruuk, and D. H. Nussey. 2010. An ecologist's guide to the animal model. J. Anim. Ecol. 79:13–26.
